# Investigating the Microbial Dynamics of *Hermetia illucens* Powder Throughout Rearing and Processing: An Integrated Approach Using Cultural and Metabarcoding Methods

**DOI:** 10.3390/foods14132161

**Published:** 2025-06-20

**Authors:** Boris Misery, Lenaïg Brulé, Rima Djema, Xin Yan, Victoire Le Cozic, Guillaume Baudouin, Michel Federighi, Géraldine Boué

**Affiliations:** 1 Oniris, INRAE, SECALIM, 44300 Nantes, France; lenaig.brule@oniris-nantes.fr (L.B.); rima.djema75@gmail.fr (R.D.); xin.yan@oniris-nantes.fr (X.Y.); victoire.le-cozic@oniris-nantes.fr (V.L.C.); 2 Cycle Farms, 6 Boulevard des Entrepreneurs, 49250 Beaufort en Anjou, France; guillaume.baudouin@cyclefarms.com; 3 Laboratoire de Sécurité des Aliments, Anses, 94700 Maisons-Alfort, France; michel.federighi@vet-alfort.fr; 4 Department of Animal Production and Public Health, Ecole Nationale Vétérinaire d’Alfort, 94700 Maisons-Alfort, France

**Keywords:** Black soldier fly, edible insect, food safety, microbiota, microbial diversity, microbial quality

## Abstract

The increasing demand for sustainable protein sources highlights *Hermetia illucens* (Black Soldier Fly, BSF) as a promising alternative. However, microbiological safety remains a key concern. This study investigated the microbial diversity of BSF larvae, comparing two processing methods: (1) boiling followed by drying and (2) drying alone. Microbial diversity was assessed via 16S rRNA sequencing, while bacterial loads were quantified using culture-based methods on samples from a French company. A systematic review complemented this analysis by synthesizing the existing knowledge on BSF microbiota. The rearing conditions varied, with substrate pH ranging from 4.1 to 9.0 and ambient temperatures between 24.6 °C and 42.7 °C. Mesophilic bacteria, spores, and lactic acid bacteria reached up to 8.6, 7.7, and 8.5 log CFU/g in the substrates and larvae, while yeasts, molds, and sulfite-reducing bacteria remained below 4.8 log CFU/g. Boiling reduced most loads below detection thresholds, particularly for yeasts, molds, and ASR. *Salmonella*, *Listeria monocytogenes*, *Cronobacter* sp., and coagulase-positive staphylococci were absent, whereas *Clostridium perfringens* and *Escherichia coli* were variably detected. Metabarcoding showed shifts in composition, with Proteobacteria, Bacteroidota, Actinobacteriota, and Firmicutes (Bacillota and Clostridiota) dominating. Process 1 more effectively reduced the bacterial loads, though *Bacillus* and *Clostridium* remained. *Campylobacter* sp. detection in powders raises food safety concerns.

## 1. Introduction

The increasing global demand for animal proteins is expected to continue until 2050, raising concerns about the sufficiency of traditional sources and highlighting the need to explore alternatives [1,2,3,4]. To address the need for sustainable food sources and to mitigate the environmental impacts of conventional livestock farming, entomophagy, the practice of consuming insects, presents a promising solution that is gaining attraction in Western countries, both for human consumption and as an alternative feed for animals [5]. It is a valuable source of essential nutrients, with the protein content ranging from 30% to 65% of their total dry matter, and it is abundant in micronutrients, such as iron, zinc, vital amino acids, and other important vitamins and minerals [6]. In addition, insect farming has a significantly lower environmental impact compared to traditional livestock production. Insects require less food and water, occupy less space, and demonstrate superior biomass conversion rates [7].

However, insects naturally harbor diverse microbial communities, including bacteria, virus, yeasts, and fungi, which play pivotal roles in their development, digestion, and overall health [8,9,10]. Additionally, insects may also carry unwanted microorganisms, including foodborne pathogens [11]. Kooh et al. (2019) and Garofalo et al. (2019) [12,13] presented the pathogenic and toxigenic microorganisms that could potentially be transmitted via edible insects, including bacteria such as *Salmonella* sp., STEC (Shiga toxin *E. coli*), *L. monocytogenes*, *C. perfringens*, *S. aureus*, and the *B. cereus* group; viruses such as Norovirus and Hepatitis Virus A; yeasts; and molds, and some studies have examined the microbial communities of edible insects [14,15,16,17,18,19,20,21]. To ensure the safety of insect-based food products, it is crucial to understand the dynamics of microbial communities during the rearing and manufacturing process of insect biomass.

Several factors influence the composition and diversity of microbial communities associated with insects, including insect species, diet, environment, and processing methods employed during biomass transformation [20,22,23]. Three main practices exist: consuming whole insects, processing them into powders or pastes, and extracting specific components, such as protein isolates and oils [24]. Processing steps such as boiling and drying have been shown to reduce microbial loads but residual spore-forming bacteria may persist [16,25]. Moreover, processing parameters such as temperature, duration, and storage conditions can affect microbial dynamics, influencing both product quality and safety [9,26,27,28]. Additional findings from Adamek et al. (2018) indicated that the most effective method for long-term storage involves killing the edible insect with boiling water, drying them at 103 °C for 12 h, and then sealing them in airtight packaging [29].

To evaluate and mitigate potential microbial risks, it is crucial to conduct a comprehensive analysis of these microbial communities throughout the entire process of insect biomass transformation. The study of Gorrens et al. (2021) [30] primarily focused on culture-dependent methods to identify and characterize bacterial, fungal, and yeast species associated with the BSF microbiota. While culture-dependent methods offer valuable insights into lab-culturable microorganisms, these techniques reveal limitations in terms of the detection threshold or assessment of the “true” diversity (e.g., 0.1 to 10% of culturable bacteria) [31]. Recent advancements in molecular microbiology have provided powerful tools for characterizing the microbial ecosystems associated with insects. High-throughput sequencing techniques, such as metabarcoding, enable detailed analysis of microbial community structures [32]. Molecular techniques such as 16S rRNA gene sequencing, metagenomics, and metatranscriptomics are commonly used to analyze the diversity, abundance, and functional potential of microbial taxa associated with the BSF [33]. These methods have revealed previously unknown microbial species and their ecological roles within the insect, for example, a study using 16S rRNA gene sequencing found *Campylobacter* sp. as one of the prevalent species in the gut of unprocessed BSF [33]. Another study investigated the gut microbiota of BSF larvae using 16S rRNA gene sequencing to assess how the substrate used for larval growth influences microbiota composition [34]. Tegtmeier et al. (2021) conducted a study on the bacterial diversity and culturability using a broad sampling approach over several rearing cycles, combining 16S rRNA amplicon sequencing with a cultivation-dependent approach and genomic fingerprinting and testing selected strains for their ability to inhibit pathogens [35]. These techniques complement traditional culture-based methods, such as plating and biochemical assays, to provide a comprehensive understanding of microbial diversity [9].

The objective of the present study is to analyze the evolution of the microbial dynamics of *Hermetia illucens* powder considering rearing and processing steps. The microbial diversity was analyzed to identify potential biological hazards, to evaluate the impact of different processing steps (boiling and drying vs. drying alone) on product safety and quality, and to identify strategies to mitigate the risk. To achieve this, a combined approach of culture-dependent and culture-independent methods was applied, based on a series of samples collected across various stages of rearing and processing steps, in collaboration with Cycle Farms, a French company based in Angers. The company employs an optimized two-step substrate approach, where the substrate is fully replaced after 5 days of rearing. In parallel, a systematic review was conducted to synthesize the existing knowledge on the microbial diversity of *Hermetia illucens*, focusing on data obtained through non-cultural methods to compare findings obtained through metagenomics while culture-dependent results will be discussed in light of the systematic review reported by Brulé et al. (2024) [36].

## 2. Materials and Methods

### 2.1. Systematic Review of the Microbial Diversity of BSF Larvae, Substrate, and Frass

Systematic research was performed to collect and synthesize the available information on the microbial diversity of BSF, based on non-cultural methods. The PRISMA (Preferred Reporting Items for Systematic Reviews and Meta-analyses) guidelines for systematic reviews were implemented using the PubMed and ScienceDirect databases [37]. The following search was used: PubMed in the title/abstract ((“Black Sol-dier Fly” OR “*Hermetia illucens*”) AND (“microb*” OR “cultural method*” OR “sequenc*” OR “metagen*” OR “metabarcoding” OR “bacteri*” OR “profil*” OR “dynamic*”)), and Web of Science with two searches (title, abstract, and keywords: (“*Hermetia illucens*” OR “Black soldier fly”) AND (“microbiol” OR “Microbiological” OR “microbial community” OR “microbiota” OR “sequencing” OR “metagenonic” OR “metabarcoding”)) and (Title, abstract, keywords: (“*Hermetia illucens*” OR “Black soldier fly”) AND (“bacteria” OR “profile” OR “dynamic”)). The search was achieved on March 21st 2025 without time or language limitations. The software Zotero (6.0.30) was used to remove duplicates. In addition, the list of references of the selected articles was examined to identify additional articles of interest. Articles providing data with non-cultural methods in BSF larvae (raw or processed), substrate, and frass were eligible for data extraction. The primary reasons for exclusion were linked to the report of only culture-dependent results, studies focusing on waste conversion and strain isolation, or articles related only to antibiotic resistance. Three evaluators shared the screening of the articles and discussed any difference or disagreement. The screening of the articles was performed on Zotero (6.0.30). The data extraction was conducted by one evaluator and checked by a second. The data were collected and synthesized in an Excel table, including the author information, publication year, country, analytical methods, sample types (raw larvae, processed larvae, substrate, and frass), and their origins (retail, farming, and processing country). It also examined the processing methods (freezing, heat treatment, drying, and microbial inactivation), rearing conditions (temperature, humidity, and feeding substrates), sample details (number of samples, number of positives, sample weight, and detection limit), and molecular techniques (DNA/RNA extraction kit reference and targeted region). Additional data included OTUs for pathogens and the genus/species identified.

### 2.2. Experimental Design

**Figure 1:**
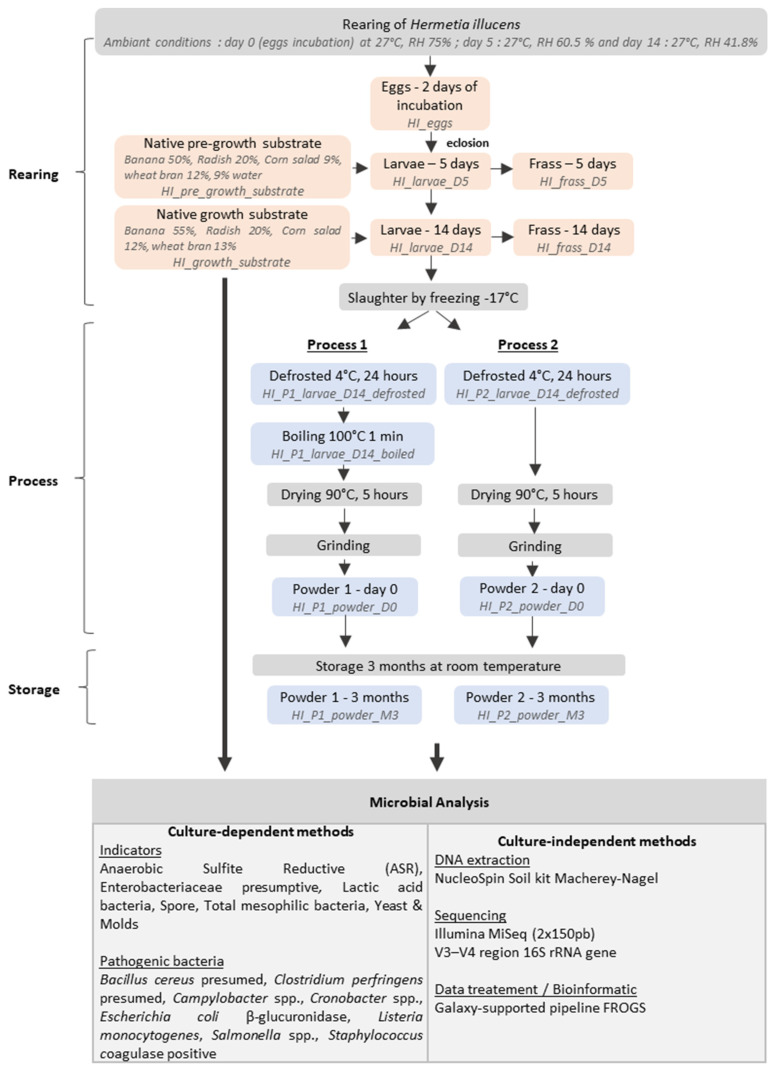
Experimental plan and sample collected.

The samples were obtained in collaboration with an industrial partner, Cycle Farms (Beaufort en Anjou, France). The rearing steps and the experimental plan are presented in Figure 1.

Cycle Farm uses a two-step substrate approach. First, a pre-growth substrate composed of 50% banana, 20% radish, 9% corn salad, 12% wheat bran, and 9% water is used for the rearing of 250,000 larvae (3rd instar, 5 days old). Due to larval size rearing constraints, the farm used five batches (dimension 60 cm × 60 cm × 12 cm) for the growth of 5000 larvae until reaching 14 days of rearing (5th instar). This growth substrate is a composition of 55% banana, 20% radish, 12% corn salad, and 13% wheat bran. The incubation occurred under controlled conditions of 27 °C and 75% relative humidity (RH) and was closely monitored and controlled with a precision of 0.1 °C and 0.1%, respectively. The data is continuously logged at hourly intervals. The specific equipment utilized for this purpose is the HB500 H egg incubator (Cimuka, Ankara, Turkey).

The eggs were collected and placed in incubators and represented the start of the rearing process. Sampling was conducted on the 5th day of rearing and on the 14th day (the time at which larvae can be processed into insect powder). For each sampling point, three technical replicates of the eggs, larvae, substrate, and frass were collected. During the rearing, a total of 1381 g of larvae (first rearing), 700 g of frass, 1000 g of pre-growth substrate, 2000 g of the growth substrate, 3276 g of larvae (second rearing), and 1081 g of frass at 5 days and 14 days, respectively, were collected. After the rearing steps, 14-day-reared larvae were killed by frosting (−17 °C) and all the samples were stored at −20 °C.

Two processes described in Figure 1 were investigated to assess the impact of heat treatments: (1) a combined boiling–drying process and (2) a drying-only process. An initial defrosted 14-day-old larvae amount (2200 g) was thawed at 4 °C for 24 h and used equally in both processes. Process 1 (P1) involved two consecutive thermal treatments (boiling and drying), while Process 2 (P2) involved a unique drying process. Before the two processes, one sample of 150 g from each process was collected for microbial enumeration and metabarcoding analysis. In Process 1, a boiling step at 100 °C for 1 min was applied, based on studies demonstrating its efficacy in inactivating non-spore-forming bacteria while preserving product quality [38,39]. In the second step, the boiled larvae were dried in an oven at 90 °C for 5 h. P2 involved a drying step in an oven at 90 °C for 5 h. During the two processes, the weight of the larvae was continuously observed and recorded every 20 min for 5 h. To evaluate the changes in the microbial communities in the insect powder after three months of storage at room temperature, the powders obtained from the larvae processed via P1 or P2 were prepared by grinding the larvae for 10 s three times using a Moulinette DPA1 (Moulinex^®^, Ecully, France). The resulting powders were divided into two batches: the first was stored at −20 °C for microbial and metabarcoding analysis immediately after processing, while the second was kept at room temperature for three months.

### 2.3. Rearing Conditions Measurement

Throughout the rearing process, the pH, temperature, and relative humidity were recorded every three days in the (pre-)growth substrates at five different locations within each rearing batch. The pH values of the rearing batch, including the larvae, substrate, and frass, were measured by a pH-meter checker HI98103 Hanna (Grosseron, France). For temperature measurement, five temperature points were selected within the tank. One temperature point was located at the center of the tank, while the remaining four points were positioned at a distance of 10 cm from each of the four corners. The relative humidity and the temperature were measured using PeakTech Temperature and Humidity Recorders (model 5185), PeakTech^®^, (Ahrensburg, Germany) according to the manufacturer’s instructions.

### 2.4. Microbial Enumeration

For each sample (except the eggs), 25 g was collected from a composite sample, homogenized from aseptically taken subsamples across different areas of the batch (e.g., top, middle, and bottom), and diluted tenfold in buffered peptone water (VWR) in a stomacher plastic bag. The samples were stomached 25.5 stroke/s for 2 min with a lab paddle blender (Masticator^®^, IUL, Barcelona, Spain). After a phase of revival of 1 h at room temperature, serial dilutions were performed in buffered peptone water up to 10^−8^. The total mesophilic flora and bacterial endospore counts were determined with the pour-plate method, respectively, on Plate Count Agar (PCA, Biokar diagnostics) or PCA with starch 2 g/L at 30 °C for 72 h after a heat treatment at 80 °C for 10 min. For the analysis of other bacterial indicators and pathogens, the samples were processed by the Eurofins laboratory using the reference methods detailed in Table 1.

### 2.5. DNA Extraction and Sequencing of Bacterial rRNA 16S Amplicons

For each sample, 2 mL was collected and centrifuged at 13,000 rpm for 10 min at 4 °C. Fourteen samples, representing distinct biological conditions across the rearing and processing workflow, were selected for DNA extraction and 16S rRNA gene sequencing. The DNA was extracted using the NucleoSpin Soil-Kit (Macherey Nagel, Hoerdt, France), following the manufacturers protocol. The DNA obtained was quantified by a NanoDrop 2000 (ThermoFischer Scientific, Illkirch-Graffenstaden, France) and was stored at −20 °C until used to send for the sequencing.

The high-throughput sequencing of the V3-V4 hypervariable region of the bacterial 16S rRNA gene of 14 samples was performed by Eurofins Genomics on an Illumina MiSeq platform according to the manufacturer’s instructions. The amplicons were generated using a two-step PCR protocol. The V3-V4 regions were PCR-amplified using template-specific primers (forward: 5′-TACGGGAGGCAGCAG-3′ and reverse: 5′-CCAGGGTATCTAATCC-3′) [40,41] containing a universal overhang. The amplicons were cleaned and set up for the PCR index, with specific primers directed to the universal overhangs. The final amplicon libraries were cleaned, quantified, and pooled equimolar. The resulting final library pool was quantified and sequenced using the v3 chemistry (2 × 300 bp paired-end reads).

### 2.6. Bioinformatic and Statistical Analyses

Paired-end reads obtained from MiSeq sequencing were analyzed using the Galaxy-supported pipeline named Find Rapidly Operational Taxonomic Units (OTUs) with Galaxy Solution (FROGS) using the dedicated guidelines for 16S data [42]. A taxonomic assignment was performed using Silva132 16S pintail 100 (bacteria, https://www.arb-silva.de, accessed on 15 November 2024). The OTU tables were used for the bioinformatic analyses. The subsequent analyses and data visualization were then performed using the R packages Phyloseq [43], DeSeq2 [44], and Ggplot2 [45]. The distribution of the sampling points (calculated from the OTU contingency table) was displayed in MDS/PCoA ordination by R software (4.2.2). Shannon, Inverse Simpson, and Chao1 estimators, along with the statistical analysis of the alpha and beta diversity indexes, used to evaluate the sample differences in species complexity, were performed using the Phyloseq R package implemented in FROGS (FROGSSTAT) [43]. Interactive sunburst plots were generated using the Plotly library v3.0.1 [46] to illustrate the relative taxonomic composition (up to the genus level) of the bacterial communities identified across the different sample types. These interactive visualizations provide hierarchical insight into microbial abundance profiles in a reader-friendly and navigable format. All the sunburst plots are available online at https://bmisery.github.io/bsf-metabarcoding-visualizations/, accessed on 3 June 2025.

## 3. Results

### 3.1. Systematic Review on the Microbial Diversity of BSF

#### 3.1.1. Study Selected

**Figure 2:**
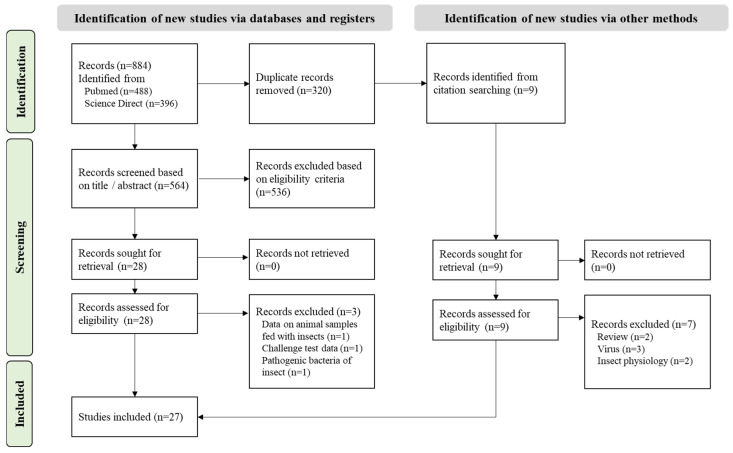
PRISMA flowchart of the systematic review on BSF microbial diversity.

A total of 680 articles were collected (Figure 2) and 564 remained after the duplicates were removed. This selection was screened based on the title, abstract, and full text, following the eligibility criteria, resulting in 27 articles selected. The data extracted are reported in Supplementary File S1.

#### 3.1.2. Study Description

**Figure 3:**
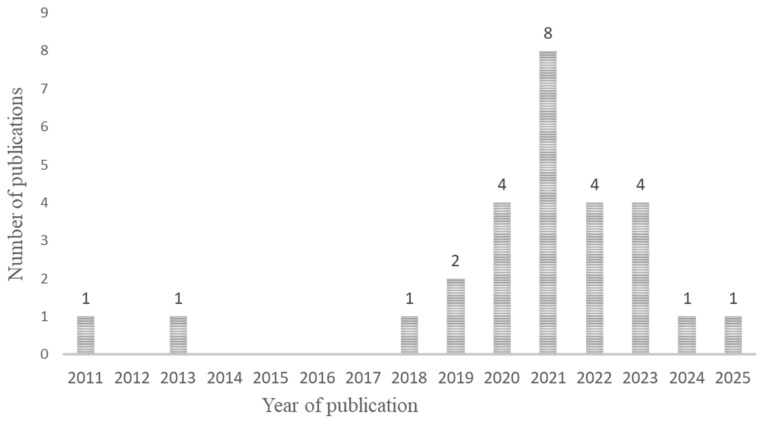
Chronological distribution of studies included in the systematic review (n = 27).

**Figure 4:**
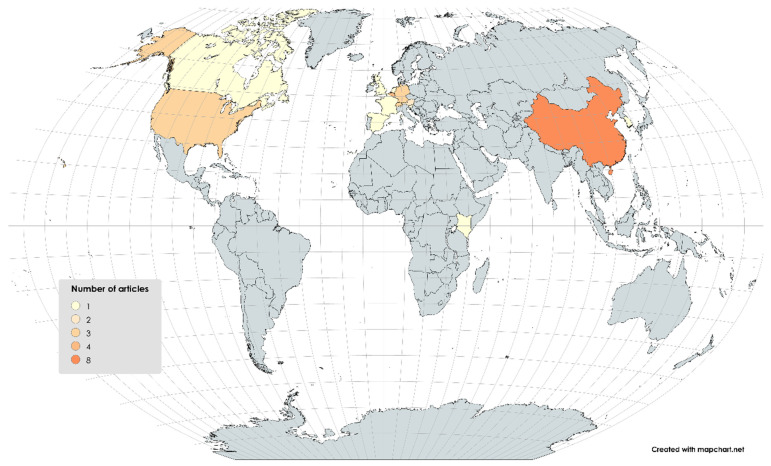
Geographical distribution of studies included in the systematic review (n = 27).

The articles found on the microbial diversity of BSF were published between 2011 and now (Figure 3) in different countries (Figure 4). The samples analyzed included mainly raw larvae (n = 27) resulting from different rearing conditions (Supplementary File S2) and some analyzed substrates (n = 13) and frass (n = 2). None of them explored the effect of the manufacturing process.

#### 3.1.3. Microbial Profile of BSF Already Published

The systematic review identified 27 studies based on non-culture-dependent methods, highlighting the extensive microbial diversity associated with *Hermetia illucens*, mainly in larvae but also in substrates and frass. While most studies focused on raw larvae from various diets and rearing conditions, none directly assessed the effect of industrial processing on microbial diversity.

High-throughput sequencing analyses (mainly 16S rRNA gene) consistently revealed the presence of bacterial genera such as *Bacillus*, *Clostridium*, *Enterococcus*, *Providencia*, *Morganella*, and *Klebsiella*, which were detected in the majority of the reviewed studies (see Table 2). These genera include spore-forming, opportunistic bacteria and potential pathogens, underscoring the importance of targeted monitoring of BSF larvae and resulting products.

Several studies, such as Gorrens et al. (2021, 2022), identified dominant communities of *Enterococcus*, *Providencia*, and *Morganella* in larvae, using extraction kits such as EZNA soil and sequencing of the V3–V4 16S rRNA regions [30,47]. Wynants et al. (2018) reported low levels of *Salmonella* and *Bacillus cereus* in larvae and/or residue samples from various European countries, suggesting that, while such pathogens may occasionally be present in raw samples, they are highly influenced by the substrate and rearing conditions [23].

Asian studies such as Ao et al. (2021) and Wu et al. (2022) also reported the persistent detection of *Providencia*, *Klebsiella*, *Morganella*, and *Dysgonomonas*, commonly associated with the larval gut and indicative of adaptation to nutrient-rich organic substrates [48,49]. Chen et al. (2023) expanded on this by combining 16S and ITS sequencing to detect not only bacteria but also molds such as *Diutina*, *Candida,* and *Issatchenkia*, depending on diet composition, emphasizing the diet’s impact on both bacterial and fungal communities [50].

Geographically, microbial profiles were broadly consistent across studies conducted in Europe (Austria, Belgium, France, Germany, Italy, Netherlands, Slovakia, Spain, and Switzerland), Asia (China and South Korea), and North America (Canada and the United States), despite differences in substrates, rearing conditions, and analytical methods. For instance, Cifuentes et al. (2020) in Germany and Bruno et al. (2019) in Italy detected *Clostridium* and *Bacillus* [8,51].

Table 2 confirms the frequent detection of *Bacillus* and *Clostridium* across the published studies. These genera were commonly found regardless of the country or method used and were detected not only in larvae but also in substrates and frass. This recurrence suggests a central ecological role for these genera in the BSF microbiome, whether as commensals or as targets for food safety monitoring.

However, several methodological limitations were identified across the existing literature. First, many studies were based on small sample sizes or lacked technical and biological replication, which limits the robustness and generalizability of the findings. Second, most investigations focused solely on taxonomic profiling using 16S rRNA sequencing, with few incorporating functional approaches, such as metabolomics, metagenomics, or metatranscriptomics. As a result, the functional roles of microbial taxa within the insect microbiome, including their potential impact on food safety or nutrient metabolism, remain largely unexplored.

In summary, the microbial profiles described in the literature reveal bacterial communities dominated by enteric Gram-negative genera (*Providencia*, *Morganella*, and *Klebsiella*), spore-forming bacteria (*Bacillus* and *Clostridium*), and Gram-positive cocci (*Enterococcus*), with the variability strongly influenced by the substrate, temperature, and rearing humidity. These findings highlight the need to establish specific microbiological criteria for insect-based products and to combine both culture-dependent and culture-independent approaches for more comprehensive microbial risk assessment.

**Table 2:** Summary of bacterial genus identified in the studies from the systematic review.

	*Acinetobacter*	*Actinomyces*	*Bacillus*	*Bacteroides*	*Burkholderia*	*Campylobacter*	*Clostridium*	*Corynebacterium*	*Dysgonomonas*	*Enterobacter*	*Enterobacteriaceae*	*Enterococcus*	*Escherichia*	*Escherichia-Shigella*	*Ignatzschineria*	*Klebsiella*	*Listeria*	*Lysinibacillus*	*Morganella*	*Paenalcaligenes*	*Proteus*	*Providencia*	*Pseudomonas*	*Rhizobiales*	*Salmonella*	*Staphylococcus*	*Vagococcus*	*Weissella*
Ao et al., 2021 [48]					X																	X		X				
Auger et al., 2023 [52]		X									X	X				X							X					
Bruno et al., 2019 [51]			X				X					X				X			X		X	X	X					
Chen et al., 2023 [50]	X		X									X				X		X										
Cifuentes et al., 2020 [53]			X	X			X									X									X			
Cifuentes et al., 2022 [8]			X								X	X							X		X	X	X					
Gorrens et al., 2021 [30]												X	X			X			X			X						
Gorrens et al., 2022 [47]			X			X	X	X			X											X				X		
Jeon et al., 2011 [54]																X			X									
Jiang et al., 2019 [55]								X														X					X	
Klammsteiner et al., 2020 [56]		X							X			X																
Klammsteiner et al., 2021 [57]	X						X							X					X									
Li et al., 2023 [34]							X		X							X						X						
Liu et al., 2020 [58]												X			X				X			X						
Mašková et al., 2025 [59]	X								X			X			X				X		X	X						X
Pei et al., 2023 [60]												X								X		X						
Querejeta et al., 2023 [61]									X				X						X		X	X						
Raimondi et al., 2020 [21]			X			X	X										X								X			
Schreven et al., 2021 [62]			X				X					X										X						
Schreven et al., 2022 [63]			X				X					X		X		X							X					
Tanga et al., 2021 [64]						X			X			X							X			X	X					
Tegtmeier et al., 2021 [35]							X					X							X		X	X						
Van Looveren et al., 2024 [65]	X											X	X						X		X	X						X
Wu et al., 2022 [49]									X	X						X			X			X						
Wynants et al., 2018 [23]			X				X					X							X						X			
Yang et al., 2021 [66]			X			X				X		X				X												X
Zheng et al., 2013 [67]			X				X																					

### 3.2. Physico-Chemical Analysis of Rearing Conditions

Table 3 presents the characteristics of the rearing pH, temperature, and relative humidity, recorded over multiple stages of insect development, including incubation, pre-growth, and growth.

The environmental and substrate conditions were closely monitored throughout rearing, with incubation maintained at 27.0 ± 0.1 °C and 75.0 ± 0.3% relative humidity; during the pre-growth phase, the substrate pH increased from 5.2 to 7.3, the internal temperature rose from 24.7 ± 0.09 °C to 42.7 ± 0.97 °C, and the humidity rose from 52.7 ± 8.4% to 63.5 ± 8.8%, while in the growth phase, the pH ranged from 4.1 to 9.0, internal temperatures from 24.6 ± 0.43 °C to 32.1 ± 0.81 °C, and humidity from 36.2 ± 2.1% to 44.2 ± 2.8%, with rearing room conditions remaining stable between 26.8 and 28.0 °C and 36–75% relative humidity.

### 3.3. Quantification of Microbial Indicators and Pathogens

**Figure 5:**
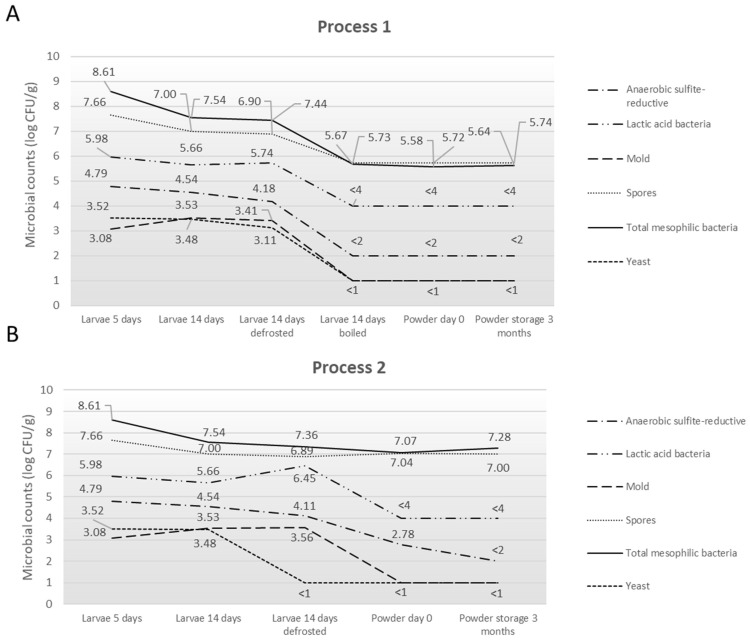
Summary of microorganism levels found in larvae and resulting powders following (**A**) Process 1 and (**B**) Process 2.

The quantification results of the microbial counts of the bacterial indicators in the larvae and resulting powders (Figure 5) as well as the frass and substrate (Table 4) highlight the microbial diversity and evolution in the total mesophilic bacteria, spores, lactic acid bacteria, yeast, mold, and anaerobic sulfite-reductive bacteria.

In the pre-growth and growth substrates, mesophilic bacteria ranged from 7.19 to 7.86 log CFU/g and spores from 4.97 to 5.39 log CFU/g, and lactic acid bacteria reached 8.48 log CFU/g in both substrates. Yeasts and molds were quantified between 2.56 and 4.60 log CFU/g and 2.60 to 3.60 log CFU/g, respectively. In larvae at D5, the counts reached 8.61 log CFU/g for mesophilic bacteria, 7.66 log CFU/g for spores, 5.98 log CFU/g for lactic acid bacteria, 3.52 log CFU/g for yeasts, and 3.08 log CFU/g for molds. At D14, the values decreased to 7.54 log CFU/g for mesophilic bacteria and 7.00 log CFU/g for spores, while lactic acid bacteria were measured at 5.66 log CFU/g. Yeasts and molds were quantified at 3.48 and 3.53 log CFU/g, respectively. Anaerobic sulfite-reducing bacteria (ASR) ranged from 4.54 to 4.79 log CFU/g. After defrosting, mesophilic bacteria were quantified at 7.44 log CFU/g, spores at 6.90 log CFU/g, lactic acid bacteria at 5.74 log CFU/g, yeasts at 3.11 log CFU/g, molds at 3.41 log CFU/g, and ASR at 4.18 log CFU/g, showing levels comparable to those measured at day 14. Following the boiling step, mesophilic bacteria were measured at 5.67 log CFU/g, spores at 5.73 log CFU/g, lactic acid bacteria at <4 log CFU/g, yeasts and molds at <1 log CFU/g, and ASR at <2 log CFU/g. The powder samples showed similar values, with mesophilic bacteria and spores around 5.58–5.74 log CFU/g and lactic acid bacteria stable at 4 log CFU/g. Yeasts, molds, and ASR remained below detection thresholds (<1 or <2 log CFU/g) during storage.

The quantification of pathogenic bacteria provided insight on *Bacillus cereus* presumed, *Clostridium perfringens* presumed, *Cronobacter* sp., *Enterobacteriaceae* presumed, *Listeria monocytogenes*, *Salmonella* sp., and *Staphylococcus* coagulase-positive (Table 5).

The results obtained highlighted that presumptive *Bacillus cereus*, *Clostridium perfringens*, *Cronobacter* sp., presumptive *Enterobacteriaceae*, *Listeria monocytogenes*, *Salmonella* sp., and coagulase-positive staphylococci were not quantified or detected in any of the sample types analyzed using culture-dependent methods. *Clostridium perfringens* was only detected in the larval sample at D5, with counts up to 4.28 CFU/g. *E. coli* was not detected in the pre-growth and growth substrates (<1 log CFU/g) but was found at 2.85 log CFU/g for larvae at D5 and 2.52 log CFU/g at D14. The frass samples showed *E. coli* contamination levels of 3.51 log CFU/g at D0 and <1 log CFU/g at D14*. E. coli* contamination in insect powders was below the quantification threshold (<1 log CFU/g) after Processes 1 and 2 and during storage.

### 3.4. Distribution of Global Phylum Taxonomic

**Figure 6:**
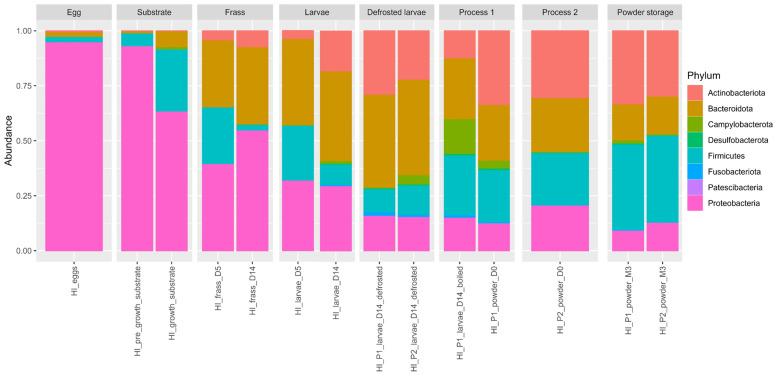
Global phylum taxonomic distribution according to the type of sample.

To describe bacterial diversity, the high-throughput sequencing of the V3–V4 hypervariable region of the bacterial 16S rRNA gene came to a total of 885,675 sequences with an average length of 300 nts (length filter with minimum 100 nts and maximum 580 nts). The sequence abundance and presence were filtered in multiple samples. The sequence proportion in the samples was kept at a minimum of 0.0005% and the Amplicon Sequence Variants (ASVs), present in at least three samples, were preserved. In this study, 84 families, 184 genera, and 362 species were detected and the final ASV table contained 599 bacterial ASVs. Figure 6 illustrates the relative abundance of the microbial phyla across the different sample types, providing a comparative overview of the taxonomic composition.

The predominant bacterial taxa in the samples were Proteobacteria and Bacteroidota, each accounting for approximately 32.42% and 24.83% of the total bacterial composition, respectively. Firmicutes (according to classical taxonomy) represented 21.23% of the overall bacterial distribution, while Actinobacteriota accounted for 5.91%. According to the revised taxonomy proposed by the Genome Taxonomy Database (GTDB), Firmicutes are now split into several phyla, mainly Bacillota and Clostridiota. In our dataset, the dominant genera formerly grouped under Firmicutes—such as *Bacillus* sp., *Enterococcus* sp., *Lactococcus* sp., *Latilactobacillus* sp., and *Paenibacillus* sp.—are now classified within Bacillota, while *Clostridium* sp., *Lachnoclostridium* sp., and *Sedimentibacter* sp. belong to Clostridiota. *Campylobacter*ota constituted a smaller proportion (0.46%) of this community. The remaining bacterial taxa represent 15.55% of the composition.

At the phylum level, the egg samples were dominated by Proteobacteria (95.01%), followed by Firmicutes (2.45%), Bacteroidota (2.10%), and Actinobacteriota (0.44%). The substrate samples were similarly dominated by Proteobacteria (80.68%), followed by Firmicutes (15.26%) and Bacteroidota (3.58%). In the frass, Proteobacteria (47.73%), Bacteroidota (32.93%), Firmicutes (13.36%), and Actinobacteriota (5.78%) were most represented. In the larvae samples, the most represented phyla were Bacteroidota (41.32%), Actinobacteriota (17.55%), Proteobacteria (23.96%), and Firmicutes (14.59%). In the boiled larvae, Bacteroidota and Firmicutes accounted for 27.66% and 27.11%, respectively, followed by Campylobacterota (15.84%), Proteobacteria (15.22%), and Actinobacteriota (12.31%). In the powder samples, Actinobacteriota ranged from 30.15% to 33.38%, Firmicutes from 30.03% to 31.07%, Bacteroidota from 20.98% to 21.54%, Proteobacteria from 10.99% to 17.55%, and Campylobacterota from 0.42% to 2.55%. Fusobacteriota, Patescibacteria, and Desulfobacterota were present at ≤0.03%.

### 3.5. Taxonomic Identification of Potential Pathogenic Bacteria

**Figure 7:**
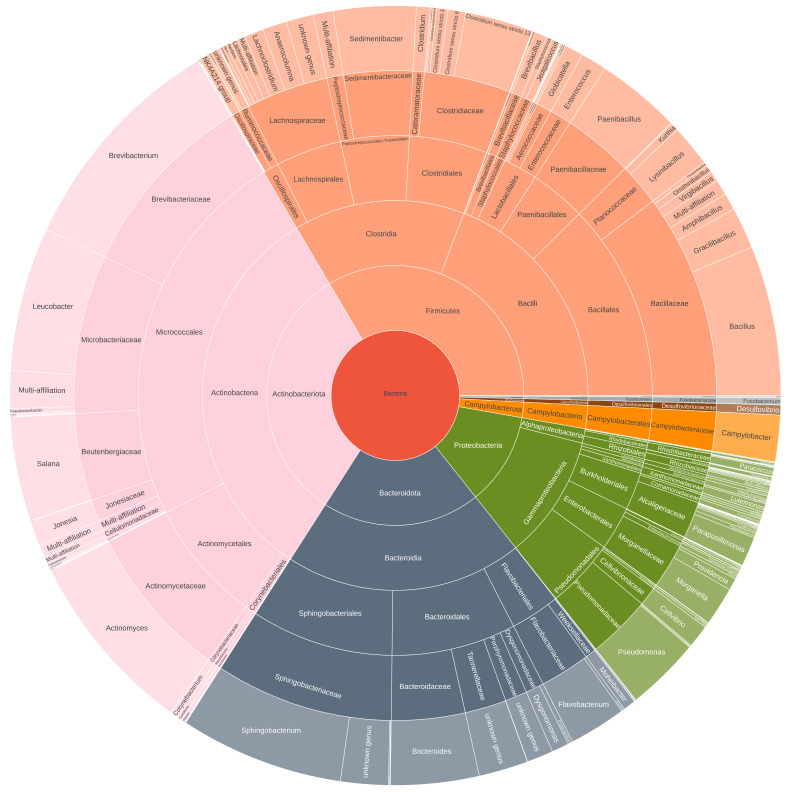
Microbial composition of the larvae powder and the presence of potential pathogens.

The microbial composition across various samples, including substrate, egg, frass, larvae, defrosted larvae, processing steps, and powder storage, provides valuable insights into the presence of potential pathogens, as shown in Figure 7 and Supplementary File S3.

In our study, the main potential pathogens detected through the 16S metabarcoding approach were *Bacillus* sp., *Campylobacter* sp., *Staphylococcus* sp., *Acinetobacter* sp., *Clostridium* sp., and *Escherichia-Shigella* sp. No *Salmonella* sp., *Cronobacter* sp., or *Listeria monocytogenes* were detected by this approach in our study.

In the substrate samples, the presence of *Campylobacter* sp. and *Staphylococcus* sp. was not detected in either the pre-growth or growth substrate samples. *Bacillus* sp. was observed at 0.01% in the pre-growth substrate and not detected in the growth substrate. *Clostridium* sp. abundance was at 0.19% in the pre-growth substrate and 0.12% in the growth substrate. *Enterobacteriaceae* species (*Escherichia* and *Shigella* sp.) were present at 0.04% in the pre-growth substrate and absent in the growth substrate. *Acinetobacter* sp. abundance was 1.10% in the pre-growth substrate and 2.63% in the growth substrate.

For the egg sample, *Campylobacter* sp. was not detected, while *Bacillus* sp. was at 0.99%. *Staphylococcus* sp. was found at 0.01%, and *Clostridium* sp. was present at 0.02%. *Enterobacteriaceae* species (*Escherichia* and *Shigella* sp.) were at 0.02%, and *Acinetobacter* sp. was detected at 0.20%.

In the frass samples, *Campylobacter* sp. was not detected at D5 but was present at 0.26% on D14. *Bacillus* sp. abundance decreased from 6.35% on D5 to 0.17% on D14. *Staphylococcus* sp. abundance remained low but present at 0.02% (D5) and 0.01% (D14). *Clostridium* sp. was consistently low at 0.04% on both days. *Enterobacteriaceae* species (*Escherichia* and *Shigella* sp.) were present at 0.24% on D5 and 0.10% on D14. *Acinetobacter* sp. abundance showed a drop from 4.43% on D5 to 0.27% on D14.

In the larvae samples, *Campylobacter* sp. showed an increase from 0.20% on D5 to 1.24% on D14. *Bacillus* sp. decreased from 4.22% on D5 to 0.54% on D14, while *Staphylococcus* sp. remained at low levels (1.09% on D5 and 0.13% on D14). *Clostridium* sp. was present at 0.30% on D5 and increased to 0.61% on D14. *Enterobacteriaceae* species (*Escherichia* and *Shigella* sp.) abundance ranged from 0.51% on D5 to 5.06% on D14. *Acinetobacter* sp. abundance was 0.62% on D5 and dropped to 0.02% on D14.

For the defrosted larvae, *Campylobacter* sp. was detected at 0.37% in P1 and 4.25% in P2. *Bacillus* sp. was observed at 1.20% in both P1 and P2. *Staphylococcus* sp. remained at low levels, with 0.18% in P1 and 0.11% in P2. *Clostridium* sp. was found at 1.17% in P1 and 1.64% in P2. *Enterobacteriaceae* species (*Escherichia* and *Shigella* sp.) abundance ranged from 1.21% in P1 to 2.46% in P2. *Acinetobacter* sp. was present at 0.05% in P1 and 0.04% in P2.

In Process 1 (boiling and drying step), *Campylobacter* sp. was found at 15.84%, and *Bacillus* sp. abundance was at 4.52%. *Staphylococcus* sp. was present at 0.11%, and *Clostridium* sp. increased to 6.55%. *Enterobacteriaceae* species (*Escherichia* and *Shigella* sp.) were present at 0.43%, and *Acinetobacter* sp. was not detected at the end of this process.

For Process 2 (drying step), *Campylobacter* sp. was also present at 3.55%, and *Bacillus* sp. remained at 3.82%. *Staphylococcus* sp. stayed at low levels (0.15%), and *Clostridium* sp. was found at 2.88%. *Enterobacteriaceae* species (*Escherichia* and *Shigella* sp.) dropped to 0.29%, and *Acinetobacter* sp. remained low at 0.17%.

In powder storage after Process 1, *Campylobacter* sp. was present at 3.55% on D0 and decreased to 1.51% after 3 months of storage (M3). *Bacillus* sp. rose from 3.82% on D0 to 8.11% on M3. *Staphylococcus* sp. abundance remained at a low level (0.15% on D0 and 0.24% on M3). *Clostridium* sp. increased from 2.88% on D0 to 5.70% on M3. *Enterobacteriaceae* species (*Escherichia* and *Shigella* sp.) were low at 0.29% on D0 and decreased to 0.14% on M3. *Acinetobacter* sp. abundance dropped from 0.17% on D0 to 0.02% on M3.

In powder storage after Process 2, *Campylobacter* sp. was present at 0.40% on D0 and 0.45% after 3 months of storage (M3). *Bacillus* sp. rose from 4.53% on D0 to 9.10% on M3. *Staphylococcus* sp. remained low at 0.22% on D0 and 0.37% on M3. *Clostridium* sp. increased from 3.86% on D0 to 6.47% on M3. *Enterobacteriaceae* species (*Escherichia* and *Shigella* sp.) were very low at 0.29% on D0 and decreased to 0.12% on M3. *Acinetobacter* sp. abundance was from 0.09% on D0 to 0.04% on M3.

### 3.6. Alpha Diversity Analysis

**Figure 8:**
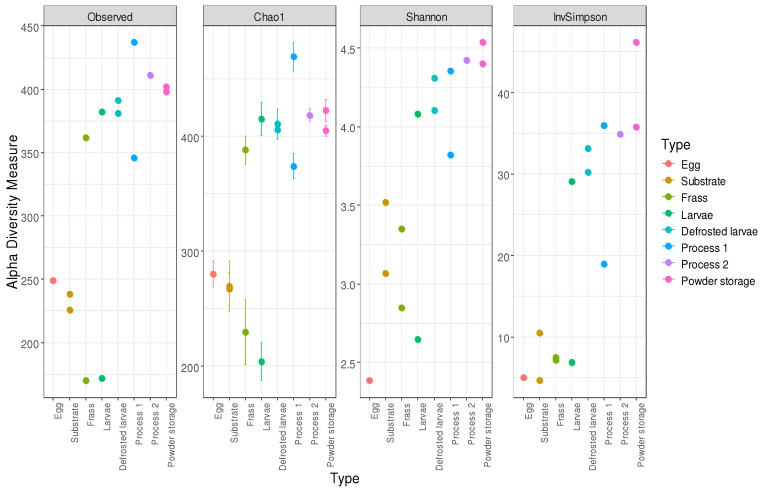
Bacterial alpha diversity measurement according to sample types.

Figure 8 illustrates the diversity indices (Observed, Chao1, Shannon, and InvSimpson) across the various sample types, providing a comprehensive comparison of the microbial diversity within the different sample groups. The Chao1 index reflected the microbial community richness and the InvSimpson and Shannon indexes reflected the microbial community diversity.

The alpha diversity metrics varied across the sample types. In the egg sample, the observed richness was 249, the Chao1 was 280.1, the Shannon index was 2.39, and the Inverse Simpson index was 5.05. The pre-growth substrate showed an observed richness of 238, a Chao1 of 267.1, a Shannon index of 3.07, and an Inverse Simpson index of 4.65. The growth substrate had an observed richness of 226, a Chao1 of 269.5, a Shannon index of 3.52, and an Inverse Simpson index of 10.49. For the frass, the values increased from D5 (observed richness 170, Chao1 229.5, Shannon index 2.85, and Inverse Simpson index 7.12) to D14 (observed richness 362, Chao1 388.1, Shannon index 3.35, and Inverse Simpson index 7.54). For the larvae, the D5 values were observed richness 172, Chao1 203.9, Shannon index 3.31, and Inverse Simpson index 11.78, while the D14 values were observed richness 382, Chao1 415.2, Shannon index 4.08, and Inverse Simpson index 29.1. In Process 1, the defrosted larvae had observed richness 391, Chao1 406.1, Shannon index 4.31, and Inverse Simpson index 33.15. The boiled larvae showed observed richness 346, Chao1 374.2, Shannon index 3.82, and Inverse Simpson index 18.95. The powder at D0 presented observed richness 437, Chao1 469.3, Shannon index 4.36, and Inverse Simpson index 35.94. In Process 2, the defrosted larvae had observed richness 381, Chao1 411.0, Shannon index 4.10, and Inverse Simpson index 30.22. The powder at D0 showed observed richness 411, Chao1 418.6, Shannon index 4.27, and Inverse Simpson index 32.06. At M1, the values were observed richness 402, Chao1 422.7, Shannon index 4.40, and Inverse Simpson index 30.38; at M3, they were observed richness 398, Chao1 405.1, Shannon index 4.54, and Inverse Simpson index 36.33.

The ANOVA performed on the Chao’s alpha, Shannon’s diversity, and Inverse Simpson’s indices revealed differences in the bacterial composition between the larvae (processed and unprocessed) and frass samples at D14 compared to D5 (*p*-value < 0.005). The ANOVA results also indicate that there is significant effect of the sample type (egg, substrate, frass, and larvae) on the observed species count (*p*-value < 0.005). The performance of the ANOVA test on the Shannon and Chao’s indexes highlighted a similar bacterial composition between the larvae (processed and unprocessed) and the insect powder after 3 months of storage.

### 3.7. Abundance of Microbial Communities

The microbial composition was analyzed using 16S rRNA gene sequencing to identify and characterize the bacterial communities in *Hermetia illucens* larvae during rearing and two distinct processing methods, referred to as Process 1 and Process 2. The microbial diversity across the rearing and processing stages of *Hermetia illucens* larvae is shown in Supplementary File S4.

The substrates used for the rearing *Hermetia illucens* larvae were characterized by a high abundance of *Enterobacter* sp., *Latilactobacillus* sp., and *Enterococcus* sp. In the pre-growth substrate, *Enterobacter* sp. dominated the bacterial community, representing approximately 50.60%, followed by *Comamonas* sp. (3.11%), *Enterococcus* sp. (1.96%), *Pseudomonas* sp. (2.15%), and *Latilactobacillus* sp. (0.38%). In the growth substrate, *Enterobacter* sp. remained predominant at 28.87%, with *Pseudomonas* sp. (3.85%), *Comamonas* sp. (3.05%), and *Dysgonomonas* sp. (4.53%) also present. Additionally, the growth substrate showed a higher abundance of *Latilactobacillus* sp. (19.90%) compared to the pre-growth substrate.

The most abundant genus in the eggs was *Pseudomonas* sp. (76.20%), followed by *Enterobacter* (5.23%), *Comamonas* sp. (1.48%), *Bacillus* sp. (0.98%), and *Enterococcus* sp. (0.11%).

In the frass samples, *Sphingobacterium* sp. and *Comamonas* sp. were the dominant genera. *Sphingobacterium* sp. accounted for 27.16% at day 5 (D5) and increased to 30.45% at day 14 (D14), while *Comamonas* sp. decreased from 31.52% at D5 to 2.42% at D14. *Pseudomonas* sp. became more prominent at D14, comprising 32.94% of the bacterial community, up from 0.27% at D5. *Bacillus* sp. was present at 6.34% at D5 but decreased to 0.17% at D14. Other bacteria detected in the frass at D14 included *Leucobacter* sp. (0.84%), *Salana* sp. (0.45%), *Actinomyces* sp. (2.11%), *Brevibacterium* sp. (0.26%), and *Campylobacter* sp. (0.26%).

At day 5, the larvae exhibited a diverse bacterial community, including *Sphingobacterium* sp. (39.15%), *Comamonas* sp. (27.09%), *Enterococcus* sp. (15.53%), *Bacillus* sp. (4.22%), *Leucobacter* sp. (0.96%), and *Campylobacter* sp. (0.20%). At day 14, the bacterial community exhibited increased diversity, with dominant genera including *Sphingobacterium* sp. (17.89%), *Actinomyces* sp. (6.00%), and *Morganella* sp. (7.99%), followed by Tannerellaceae species (7.31%), and *Porphyromonadaceae* (8.74%). Other genera present were *Brevibacterium* sp. (3.58%), *Leucobacter* sp. (2.37%), *Enterococcus* sp. (3.93%), *Salana* sp. (1.86%), *Campylobacter* sp. (1.24%), and *Dysgonomonas* sp. (3.59%). *Bacteroides* sp. was also detected at 0.80%.

The microbial composition of *Hermetia illucens* larvae was analyzed during the two processing methods, Process 1 and Process 2, to assess the dynamic changes in the bacterial communities across the different stages of processing.

The microbial communities of the larvae at day 14 post-defrosting for Process 1 and Process 2 shared many similarities but with varying abundances. Both samples contained *Sphingobacterium* sp. (13.11–23.85%), Tannerellaceae sp. (5.68–13.86%), *Salana* sp. (3.25–5.36%), *Dysgonomonas* sp. (3.37–3.91%), *Leucobacter* sp. (2.38–3.23%), *Morganella* sp. (1.93–6.72%), *Bacillus* sp. (1.20% in both), *Escherichia-Shigella* sp. (1.21–2.46%), *Paracoccus* sp. (0.80–1.88%), and *Fusobacterium* sp. (0.97–1.62%). The differences in the abundance between the two processes included *Campylobacter* sp., which is more abundant in Process 2 (4.25%) compared to Process 1 (0.37%); *Morganella* sp. (6.72% in Process 2, 1.93% in Process 1); and Tannerellaceae sp. (13.86% in Process 2, 5.68% in Process 1).

In Process 1, boiled larvae at day 14 (D14) and powdered larvae at day 0 (D0) and after three months (M3) showed a dynamic shift in the bacterial community. For the boiled larvae, *Campylobacter* sp. was the dominant genus, comprising 15.84% of the bacterial community, followed by Tannerellaceae sp. (6.26%), *Actinomyces* sp. (4.77%), and *Salana* sp. (4.14%). Other genera included *Bacillus* sp. (4.52%), *Dysgonomonas* sp. (1.84%), *Enterococcus* sp. (2.81%), *Sphingobacterium* sp. (1.74%), *Brevibacterium* sp. (1.02%), *Flaviflexus* sp. (0.48%), *Corynebacterium* sp. (0.32%), and *Lysinimicrobium* sp. (0.26%). The powdered larvae at D0 showed *Brevibacterium* sp. (10.18%), *Leucobacter* sp. (9.81%), and *Sphingobacterium* sp. (9.41%) as the most abundant genera. Other genera present included *Actinomyces* sp. (7.10%), *Comamonas* sp. (3.55%), *Bacillus* sp. (3.82%), *Flavobacterium* sp. (2.70%), *Bacteroides* sp. (2.44%), Tannerellaceae sp. (2.54%), *Dysgonomonas* sp. (0.84%), and *Campylobacter* sp. (0.88%). After 3 months of storage (M3), *Leucobacter* sp. (7.32%), *Sphingobacterium* sp. (7.20%), and *Bacillus* sp. (8.11%) remained the most prominent genera. *Brevibacterium* sp. (12.34%) and *Actinomyces* sp. (6.44%) were still present, along with *Enterococcus* sp. (1.37%). Other genera such as *Salana* sp. (4.07%), *Pseudoclavibacter* sp. (1.08%), and *Dysgonomonas* sp. (0.60%) were also found.

In Process 2, the powdered larvae at D0 and after three months of storage (M3) displayed distinct microbial profiles. At D0, the community was dominated by *Sphingobacterium* sp. (15.07%), followed by *Leucobacter* sp. (6.23%), *Brevibacterium* sp. (5.40%), and *Bacillus* sp. (4.53%). Other genera included *Dysgonomonas* sp. (1.37%), *Lysinibacillus* sp. (1.06%), and *Enterococcus* sp. (0.99%). Also present were Tannerellaceae sp. (2.05%), *Amphibacillus* sp. (0.52%), and *Gordonia* sp. (0.25%). After three months of storage, the microbial community of the powdered larvae in Process 2 remained dominated by *Bacillus* sp. (9.10%) and *Sphingobacterium* sp. (10.12%), while *Lysinibacillus* sp. (2.30%) increased and *Leucobacter* sp. (4.76%) decreased compared to their levels at D0. *Enterococcus* sp. (1.52%) was also detected, along with *Gracilibacillus* sp. (0.43%), *Moheibacter* sp. (0.34%), and *Lysinimicrobium* sp. (0.67%). *Dysgonomonas* sp. (0.90%), Tannerellaceae sp. (2.26%), and *Amphibacillus* sp. (1.10%) were also present.

### 3.8. Comparison of Microbial Diversity (Process/Rearing)

**Figure 9:**
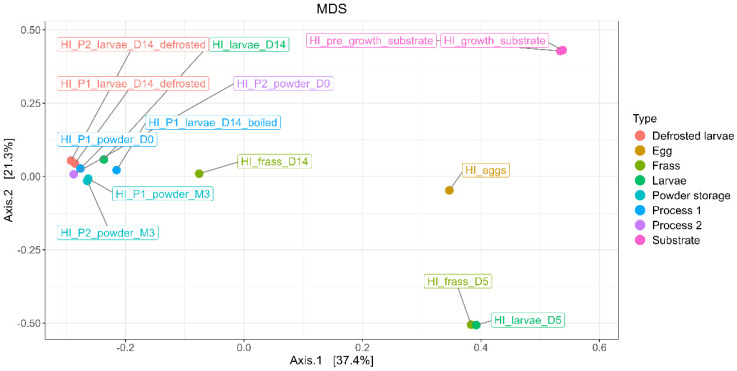
MDS/PCoA representing bacterial ASV distribution according to the type of samples (egg, substrate, frass, larvae, processing, and powder).

To examine the differences in the bacterial composition between the sample types, the microbial profiles were analyzed using MDS/PCoA, providing a visual representation of the distribution of bacterial ASVs across the different categories (egg, substrate, frass, larvae, processing, and powder), as shown in Figure 9.

The results show that the first axis (PC1) explains 37.4% of the variance, while the second axis (PC2) explains 21.3%, collectively accounting for 58.7% of the total variance. The bacterial composition of the egg and substrate samples was significantly distinct from the larvae and frass samples. The bacterial composition of the egg samples exhibited the most distinct clustering in the MDS/PCoA analysis, reflecting their unique microbial community. Similarly, the bacterial profiles of the substrate samples, including the pre-growth and growth substrates, were significantly different from all the other sample types, forming a distinct cluster. The bacterial compositions of the samples from day 5 (D5), including both the frass and larvae samples, showed similar microbial profiles. These samples were distinctly separated from those collected at day 14 (D14), particularly the larvae samples, which exhibited a different microbial composition.

The larvae samples at D14 (processed and unprocessed) formed a separate and distinct cluster, highlighting significant shifts in the microbial composition over time. The frass and larvae samples from D5 and D14 also exhibited clear separation, indicating dynamic changes in the bacterial communities as the rearing progressed.

**Figure 10:**
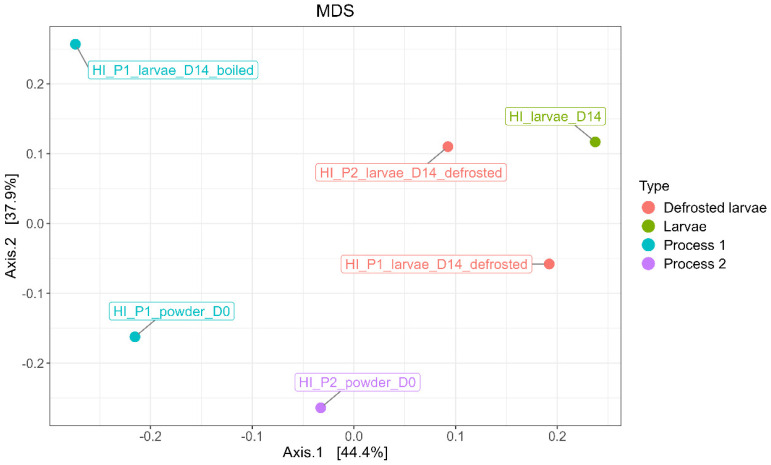
MDS/PCoA representing bacterial ASV distribution according to the type of samples (processed larvae and powder).

The MDS/PCoA plot in Figure 10 illustrates the distribution of the samples based on the abundance of the observed OTUs and shows the distribution of ASVs according to the larvae processed by Process 1 and 2, as well as the associated powder samples.

A variation in the bacterial composition between the powder samples and the larvae (both processed and unprocessed) was observed. Similarly, the boiled sample exhibited a distinct bacterial composition compared to the final powder samples and the larvae samples. The microbial communities associated with Process 1 and Process 2 also differed. No significant difference in bacterial composition was found between the powder samples at D0 and after 3 months of storage (M3).

The R packages DeSeq2 was used to underline the significant fold change in the bacterial taxa between the larvae (processed and unprocessed) and insect powder. All the differences in the microbial proportions between the tanks quoted in this paragraph correspond to a fold change > 2 with a *p-*value < 0.001. The powder samples were enriched with ASVs affiliated to *Bacillus* species (three ASVs), *Paenibacillus* sp., *Clostridium* sp., and *Anaerocolumna* sp., while the larvae samples were enriched with *Acinetobacter* sp., *Sphingobacterium* sp., *Comamonas* sp., *Enterococcus* sp., and *Enterobacteriaceae* species. We performed the same analysis comparing the larvae (processed and unprocessed) with the substrates. The substrate samples were enriched with ASVs affiliated with species from the *Enterobacteriaceae* family (e.g., *Enterobacter* sp. and *Rhanella* sp.) and *Lactobacillus* sp. (e.g., *Lactobacillus sakei*), while the larvae samples showed an enrichment of bacteria commonly associated with the gut microbiome of larvae, including *Sphingobacterium* sp., *Flavobacterium* sp., *Olivibacter* sp., *Parapedobacter* sp., Porphyromonadaceae species, *Prevotella* sp., *Dysgonomonas* sp., *Candidatus* sp., Bacteroides species, *Moheibacter* sp., *Myroides* sp., *Klebsiella* sp., *Proteus* sp., *Providencia* sp., and *Morganella* sp.

## 4. Discussion

Understanding the microbial dynamics of *Hermetia illucens* throughout rearing and processing is crucial for optimizing production methods and ensuring food safety. Our findings confirm previous research while also highlighting key differences that can be attributed to specific rearing conditions, microbial interactions, and processing methods.

The composition of the substrate played a central role in shaping the microbial communities of *H. illucens*. The pre-growth substrate was dominated by *Enterobacter* sp. and *Latilactobacillus* sp., taxa frequently associated with organic matter decomposition and lactic acid fermentation [23,63]. This microbial profile aligns with previous studies reporting that carbohydrate-rich substrates promote lactic acid bacteria, which contribute to microbial stabilization by outcompeting potential pathogens [25]. In contrast, the eggs displayed a distinct microbiota dominated by *Pseudomonas* sp. (76.2%), a genus often associated with environmental persistence and biofilm formation [51]. The presence of *Pseudomonas* sp. in eggs suggests that microbial colonization may occur early in development, possibly through vertical transmission, as proposed in other insect models [62,68]. Such early associations may contribute to the establishment of a core microbiota involved in substrate decomposition, thereby enhancing the ecological plasticity of the species [62,65,68].

As larvae developed, a notable shift in microbial diversity was observed. At day 5 (D5), the community was dominated by *Sphingobacterium* sp. and *Comamonas* sp., two genera previously identified in *H. illucens* larvae and known for their involvement in organic matter degradation [22]. By day 14 (D14), the microbiota became more diverse, with an increase in *Morganella* sp. and *Actinomyces* sp., mirroring findings from Schreven et al. (2022) that demonstrated microbiota shifts in response to larval metabolic activity and substrate composition [63]. This transition also supports previous research showing that gut microbiota diversity increases with larval age as new taxa colonize the digestive tract [23,51,69]. Unlike Schreven et al. (2022), who reported a higher prevalence of lactic acid bacteria in larvae, we observed a dominance of *Sphingobacterium* sp. and *Comamonas* sp. at early developmental stages, suggesting that microbial composition is highly dependent on rearing conditions [63]. Recent metabarcoding analysis further supports this notion, demonstrating clear stage-specific shifts in microbial diversity and composition in *H. illucens*, suggesting that microbial communities dynamically respond to environmental and nutritional inputs rather than being randomly distributed [61]. The microbial composition of frass closely resembled that of larvae, reinforcing the hypothesis that gut-associated bacteria are continuously excreted into the rearing environment, a process described in Cifuentes et al. (2020) [53]. The detection of *Bacillus* sp. and *Clostridium* sp. in frass suggests that sporulated bacteria persist throughout the rearing cycle, as also reported by Vandeweyer et al. (2017) [10].

Processing significantly altered the microbial composition of *H. illucens*, with notable differences between the two processing methods. Boiling followed by drying (Process 1) was effective in reducing microbial loads, particularly mesophilic bacteria, but had a limited effect on spore-forming bacteria, consistent with previous findings on heat-treated edible insects [24,70]. However, metabarcoding analysis revealed the persistence of *Bacillus* sp. and *Clostridium* sp. post-processing, while culture-dependent methods revealed spore counts of 5.74 CFU/g, indicating that thermal treatment alone might be insufficient to completely eradicate these resilient taxa [16]. Notably, the increased relative abundance of *Campylobacter* sp. detected in boiled larvae (15.84%) likely results from the enhanced release and detection of bacterial DNA following heat-induced cell disruption. In contrast, drying alone (Process 2) preserved a higher microbial diversity, particularly mesophilic bacteria, supporting findings by Wynants et al. (2019) that drying alone is insufficient for complete microbial inactivation [23]. While Process 2 did not select for *Campylobacter* sp., spore-forming bacteria such as *Bacillus* sp. and *Clostridium* sp. remained abundant, highlighting the need for additional decontamination strategies. Microbial diversity remained stable in the powders stored for three months, suggesting that processed powders are microbiologically resilient under controlled conditions. However, the relative abundance of *Bacillus* sp. increased during storage, a trend also noted in Fasolato et al. (2018), emphasizing the importance of optimizing storage conditions to prevent spore germination [16].

The absence of *Salmonella* sp., *Listeria monocytogenes*, and *Cronobacter* sp. aligns with previous studies, suggesting that these pathogens are not commonly associated with *H. illucens* when rearing and processing conditions are properly managed [25]. However, given the variability observed across different studies, establishing microbiological criteria specific to insect-based foods would enhance safety and ensure regulatory compliance. The identification of *Campylobacter* sp., *Bacillus* sp., and *Clostridium* sp. in the final powders raises concerns regarding food safety. *Campylobacter* sp. are major foodborne pathogens, though their role in edible insects remains underexplored. Although *Campylobacter* spp. were not detected in some previous studies on processed *H. illucens* [14,17], the occurrence of *Campylobacter* sp. in larvae samples has also been documented by Gorrens et al. (2022), who reported the consistent detection of a *Campylobacter*-like OTU across multiple insect rearing cycles, highlighting potential microbiological risks and underscoring the necessity for stringent hygiene practices during rearing and processing [47]. In our study, their detection may be linked to the presence of cells in a viable but non-culturable (VBNC) state induced by thermal stress, which can impair cultivation on selective media while allowing detection through DNA-based methods, such as metabarcoding. This finding may also reflect the presence of *Campylobacter* sp. in a viable but non-culturable (VBNC) state, a stress response known to occur after heat exposure in some *Campylobacter* species [71,72]. Previous risk assessments, including Kooh et al. (2020), suggested that *Campylobacter* sp. exhibits low resistance to processing in *T. molitor* [38]. The detection of *Campylobacter* sp. in BSF larvae aligns with prior studies demonstrating the presence and potential transmission of *Campylobacter jejuni* in other insect vectors, such as *Musca domestica* and *Alphitobius diaperinus*, which act as mechanical carriers within agricultural environments [60,73,74]. These findings contribute to the growing evidence that insects can harbor and potentially disseminate *Campylobacter* spp., reinforcing observations from previous research on house flies and lesser mealworms, where viable *Campylobacter* cells were recovered from both external surfaces and excreta, suggesting a possible role in pathogen resilience and transmission [60,74,75,76]. To better assess the presence of stressed or VBNC *Campylobacter* cells, future studies could benefit from the application of enrichment protocols such as the Park and Sanders method, which has proven effective in recovering sublethally injured cells, particularly in complex matrices [77,78].

While most *Bacillus* sp. species are not pathogenic, some within the *B. cereus* group could pose risks, particularly in products stored under suboptimal conditions [16]. Spore-forming bacteria such as *Bacillus* sp. and *Clostridium* sp. remained detectable after processing, consistent with previous reports [7,38]. The persistence of *Clostridium* sp. spores further highlights the need for additional decontamination strategies, such as high-pressure processing (HPP) or controlled humidity drying, as suggested by Osimani et al. (2021) [70]. It is crucial to acknowledge that the detection of these genera does not inherently imply the presence of pathogenic species. Numerous *Bacillus* species act as environmental decomposers and exhibit mutualistic or symbiotic relationships with insects [79]. Moreover, *Bacillus* species, commonly detected in the microbiota of *Hermetia illucens*, play significant ecological roles such as substrate decomposition and nutrient cycling, thereby promoting insect health and development [61,80]. Likewise, several *Clostridium* species are commensals in animal gastrointestinal tracts, distinct from pathogenic strains such as *C. perfringens* [81]. Recent evidence shows that intestinal *Clostridium* populations in insects may actively contribute to digestion processes, immune modulation, and the maintenance of intestinal homeostasis rather than posing a health risk [60,82]. This could explain discrepancies between culture-based and molecular detection methods, highlighting the need for further investigations into its survival mechanisms in insect-based products. These findings underscore the importance of species-specific risk assessments to optimize decontamination strategies and ensure microbial safety in edible insect production.

The culture-dependent analyses in our study did not detect *B. cereus*, *and Clostridium perfringens* was found only transiently at day 5, suggesting limitations in the applicability of traditional foodborne microbial standards designed for food matrices to insect matrices, which typically possess high microbial loads capable of obscuring targeted pathogen detection [15,36]. Indeed, insects such as *H. illucens* harbor a complex microbiome that can competitively exclude pathogens and reduce their culturability, emphasizing the need for methodological adaptations in microbial risk assessments specific to insect matrices [83]. Moreover, culture-based methods might not capture novel or less characterized pathogenic strains of *Bacillus* or *Clostridium*, emphasizing the necessity for comprehensive characterization and risk assessment of potentially pathogenic taxa in edible insects [84]. The persistence of *Bacillus* sp. and *Clostridium* sp. after processing corroborates previous research, emphasizing the necessity of targeted microbial risk management strategies in edible insect production [16,20,23]. Interestingly, while amplicon-based 16S rRNA profiling revealed the presence of these genera in post-processing samples, they were not detected using culture-dependent methods, raising questions about the viability of the detected taxa or the potential presence of non-culturable or sublethally injured cells. This discrepancy might be partly explained by the ecological role of insect-produced antimicrobial peptides (AMPs). BSF larvae naturally synthesize antimicrobial peptides (AMPs) as a critical component of their innate immune system, modulating microbial populations within their gut environment. Such peptides can inhibit or reduce the viability and culturability of sensitive bacterial taxa, thereby contributing to observed differences between culture-based and molecular detection methods [85,86,87]. Moreover, the limited taxonomic resolution of short-read 16S sequencing (e.g., V3–V4) complicates species-level identification, thereby precluding definitive assessment of pathogenicity and necessitating reference to these taxa as putative pathogenic bacteria pending further detailed characterization [88]. This underscores the need for a deeper taxonomic resolution to distinguish between innocuous and potentially hazardous species. Future research should focus on optimizing decontamination strategies, such as vacuum-assisted drying or fermentation, while leveraging full-length 16S metabarcoding and metagenomic sequencing to assess microbial dynamics and processing effects on food safety [89,90,91,92]. Full-length 16S rRNA sequencing offers superior taxonomic resolution over targeted V3-V4 regions, particularly at the species level [93], while metagenomics provides a broader perspective by detecting low-abundance taxa and revealing functional traits [92]. In parallel, fungal communities were only assessed using culture-based methods in our study. To better capture the full microbial ecology of insect matrices, future research should incorporate ITS-based sequencing to characterize fungal diversity and evaluate the impact of processing on these communities and potential safety concerns. Advances in sequencing technologies refine these methods, with Oxford Nanopore R10.4.1 and PacBio Sequel II improving species-level resolution in environmental microbiomes [94]. Additionally, optimized primer sets and bioinformatics pipelines enhance classification accuracy and mitigate PCR bias, allowing for better characterization of complex microbial communities [95,96]. Future studies should focus on overcoming methodological limitations and integrating multi-omics approaches, such as metatranscriptomics, to better assess microbial activity and its role in the safety and quality of insect-based products [33,60,97,98].

This study provides a comprehensive analysis of the microbial dynamics in *Hermetia illucens* during rearing and processing, highlighting the influence of the substrate composition, developmental stage, and processing method on microbial diversity. While thermal processing effectively reduced the overall microbial loads, certain taxa—including spore-forming bacteria such as *Bacillus* sp. and *Clostridium* sp.—remained detectable in processed samples due to their heat-resistant spores. Additionally, although *Campylobacter* sp. was not detected post-processing, the continuous detection of these microorganisms highlights the importance of developing more sensitive and targeted microbial monitoring strategies. These findings contribute to the growing body of knowledge on the microbial safety of edible insects and underscore the importance of tailored decontamination strategies for their safe commercialization. Future research should focus on refining processing parameters and developing predictive models to assess microbial risks in insect-based food production.

## 5. Conclusions

In conclusion, this study provides valuable insights into the microbiota characterization of the edible insect species *Hermetia illucens* (Black Soldier Fly). The microbial analysis revealed high microbial loads and a diverse bacterial community, including spore-forming bacteria such as *Clostridium* spp. and *Bacillus* spp. While human foodborne pathogens like *Salmonella* spp., *Listeria monocytogenes*, and *Cronobacter* spp. were not detected, the presence of certain bacterial taxa, particularly spore-forming bacteria, may represent a potential risk under specific conditions. Furthermore, this study demonstrated that processing steps significantly influence microbial reduction, with the combination of boiling and drying (Process 1) being more effective than drying alone (Process 2). Future research should investigate alternative decontamination strategies to ensure microbial safety in insect-derived products. Emerging techniques such as high-pressure processing (HPP) or controlled fermentation may offer effective solutions. Despite the growing interest in the microbiota of BSF larvae, there is still a lack of comprehensive data on the microbial safety of processed insect powders. In particular, studies combining culture-dependent and culture-independent approaches remain scarce, although such integration is essential to obtain a more complete and robust picture of microbial risks. Our results highlight the need to further explore these complementary tools, especially in the context of insect-based food products. Beyond identifying risks, this study underlines the importance of building operational solutions to support producers in controlling microbial hazards. Establishing insect-specific microbiological criteria, designing effective monitoring strategies across the production chain, and identifying critical control points adapted to insect farming are key priorities. Moreover, strengthening collaborations between researchers, food safety authorities, and insect producers is crucial to co-develop pragmatic guidelines and risk management practices. These should aim to improve both the microbial safety and the consumer acceptability of insect-based foods. As Europe moves towards the approval of insects for a potential protein alternative, particularly *Hermetia illucens*, the anticipated increase in consumption underscores the importance of further studies to assess and manage microbial risks across various processing methods.

## Figures and Tables

**Table 1:** List of microbiological analysis methods used for culture-dependent analysis.

Indicators	Methods
ASR, Anaerobic Sulfite Reductive (46 °C)	NF V08-061 boxes
*Bacillus cereus* presumed	NF EN ISO 7932
*Campylobacter* spp.	NF EN ISO 10272-2/A1
*Clostridium perfringens* presumed	NF EN ISO 7937
*Cronobacter* spp.	NF EN ISO 22964
*Enterobacteriaceae* presumed	NF V08-054
*Escherichia coli* β-glucuronidase positive	NF ISO 16649-2
Lactic acid bacteria	NF ISO 15214
*Listeria monocytogenes*	BKR 23/02-11/02
Molds	Internal method adapted (DG18, 120 h ± 3 h)
*Salmonella* spp.	BKR 23/07-10/11
*Staphylococcus* coagulase-positive	Internal method adapted of NF EN ISO 6888-2
Yeasts	Internal method adapted (NF V 08-036)

**Table 3:** Physico-chemical and environmental parameters during the different rearing phases of *Hermetia illucens*. Data are the mean of replicates ± standard deviation. * Each growth stage (pre-growth and growth) had a distinct substrate, which was changed between these two stages.

		Rearing Data Sample			Environmental Rearing Data (Room)	
	Date	pH	T°C	RH%	T°C	RH%
Incubation (eggs)	Day 0	-	-	-	27.0 ± 0.1	75.0 ± 0.3
Pre-Growth Batch *	Day 2	5.2	24.7 ± 0.09	52.7 ± 8.4	26.9 ± 0.8	52.7 ± 8.4
	Day 5	7.3	42.7 ± 0.97	63.5 ± 8.8	26.8 ± 0.5	63.5 ± 8.8
Growth batch *	Day 6	4.1	24.64 ± 0.43	43.7 ± 2.5	27.9 ± 1.7	43.7 ± 2.5
	Day 8	8.5	31.84 ± 0.64	44.2 ± 2.8	27.7 ± 1.5	44.2 ± 2.8
	Day 12	8.9	32.08 ± 0.81	36.2 ± 2.1	28.0 ± 1.2	36.2 ± 2.1
	Day 14	9.0	30.2 ± 0.47	43.6 ± 2.6	27.8 ± 2.0	43.6 ± 2.6

**Table 4:** Summary of microorganism levels (log CFU/g) found in the substrate and frass during the rearing of *Hermetia illucens*. Data are the mean of replicates ± standard deviation. ^a^,^b^, respectively, one sample of less than 2 and 4 log cfu/g.

	Anaerobic Sulfite-Reductive	*Lactic Acid Bacteria*	Mold	Spores	Total Mesophilic Bacteria	Yeast
Substrate	2.70 ^a^ ± 0.99	8.48 ± 0.00	3.10 ± 0.71	5.18 ± 0.30	7.53 ± 0.48	3.58 ± 1.45
Frass	4.72 ± 0.51	6.24 ^b^ ± 3.17	3.43 ± 0.61	7.82 ± 0.29	9.29 ± 0.61	3.36 ± 1.11

**Table 5:** Summary of microorganism levels (log cfu/g) found during the rearing of *Hermetia illucens* (substrates, larvae, and frass) and after processing (powder). Substrate and frass at days 5 and 14; larvae at day 5, 14, and defrosted; and powder at day 0 and after 3 months of storage. Data are the mean of replicates ± standard deviation or all values are reported when at least one sample was below the limit of quantification. * at least one sample less than 1 log cfu/g. ND is not detected in 25 g.

	Sample Code	*Bacillus cereus* Presumed	*Clostridium perfringens* Presumed	*Cronobacter* spp.	*Enterobacteriaceae* Presumed	*Escherichia coli* β-Glucuronidase-Positive	*Listeria monocytogenes*	*Salmonella* spp.	Staphylococci Coagulase-Positive
Substrate	HI_pre_growth_substrate HI_growth_substrate	<1	<1	Not detected	3.08 ± 0.18	<1	Not detected	Not detected	<1
Frass	HI_frass_D5, HI_frass_D14	<1	<1	Not detected	2.78 ± 1.11	<1, 2.51 *	Not detected	Not detected	<1
Larvae	HI_larvae_D5, HI_larvae_D14, HI_P1_larvae_D14_defrosted, HI_P2_larvae_D14_defrosted	<1	<1, <1, <1, 4.28 *	Not detected	3.61 ± 0.77	2.62 ± 0.26	Not detected	Not detected	<1
Boiled larvae (applied for Process 1)	HI_P1_larvae_D14_boiled	<1	<1	Not detected	<2	<1	Not detected	Not detected	<1
Powder Process 1	HI_P1_powder_D0, HI_P1_powder_M3	<1	<1	Not detected	<2	<1	Not detected	Not detected	<1
Powder Process 2	HI_P2_powder_D0, HI_P2_powder_M3	<1	<1	Not detected	<2	<1	Not detected	Not detected	<1

## Data Availability

The original contributions presented in this study are included in the article/Supplementary Materials. Further inquiries can be directed to the corresponding author.

## Supplementary Materials

The following supporting information can be downloaded at: https://www.mdpi.com/article/10.3390/foods14132161/s1, Supplementary File S1. Synthesis of samples included in the systematic review; Supplementary File S2. Characteristics and methodologies of included studies in the PRISMA systematic review on microbial risk in insect-based products; Supplementary File S3. Distribution of potentially pathogenic bacteria across different rearing and processing matrices of insects; Supplementary File S4. Phylogenetic taxonomy for bacterial communities showing dynamic changes at the genus level during *Hermetia illucens* rearing and processing.
