# Supplementary material for: Investigating the Microbial Dynamics of Hermetia illucens Powder Throughout Rearing and Processing: An Integrated Approach Using Cultural and Metabarcoding Methods
Source: Foods. 2025 Jun 20;14(13):2161. doi: 10.3390/foods14132161 (PMC12248505; doi:10.3390/foods14132161)

Supplementary file S3 Distribution of potentially pathogenic bacteria across different rearing and processing matrices of insects. All sunburst plots are available online at https://bmisery.github.io/bsf-metabarcoding-visualizations/.

S3-A Taxonomic distribution of microbial community in egg sample (HI_egg)

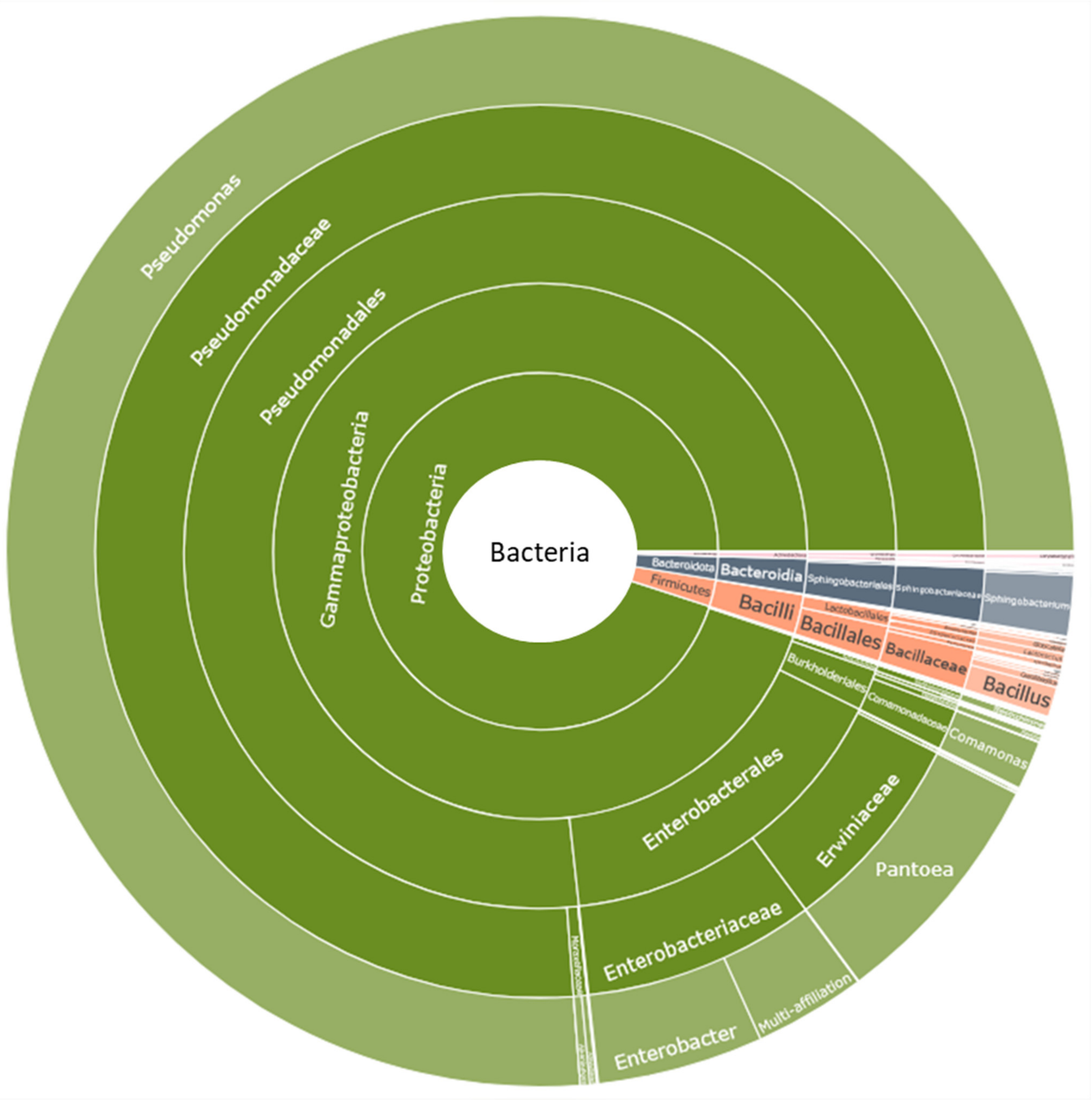

S3-B Taxonomic distribution of microbial community in substrate samples (HI_pre_growth_substrate, HI_growth_substrate)

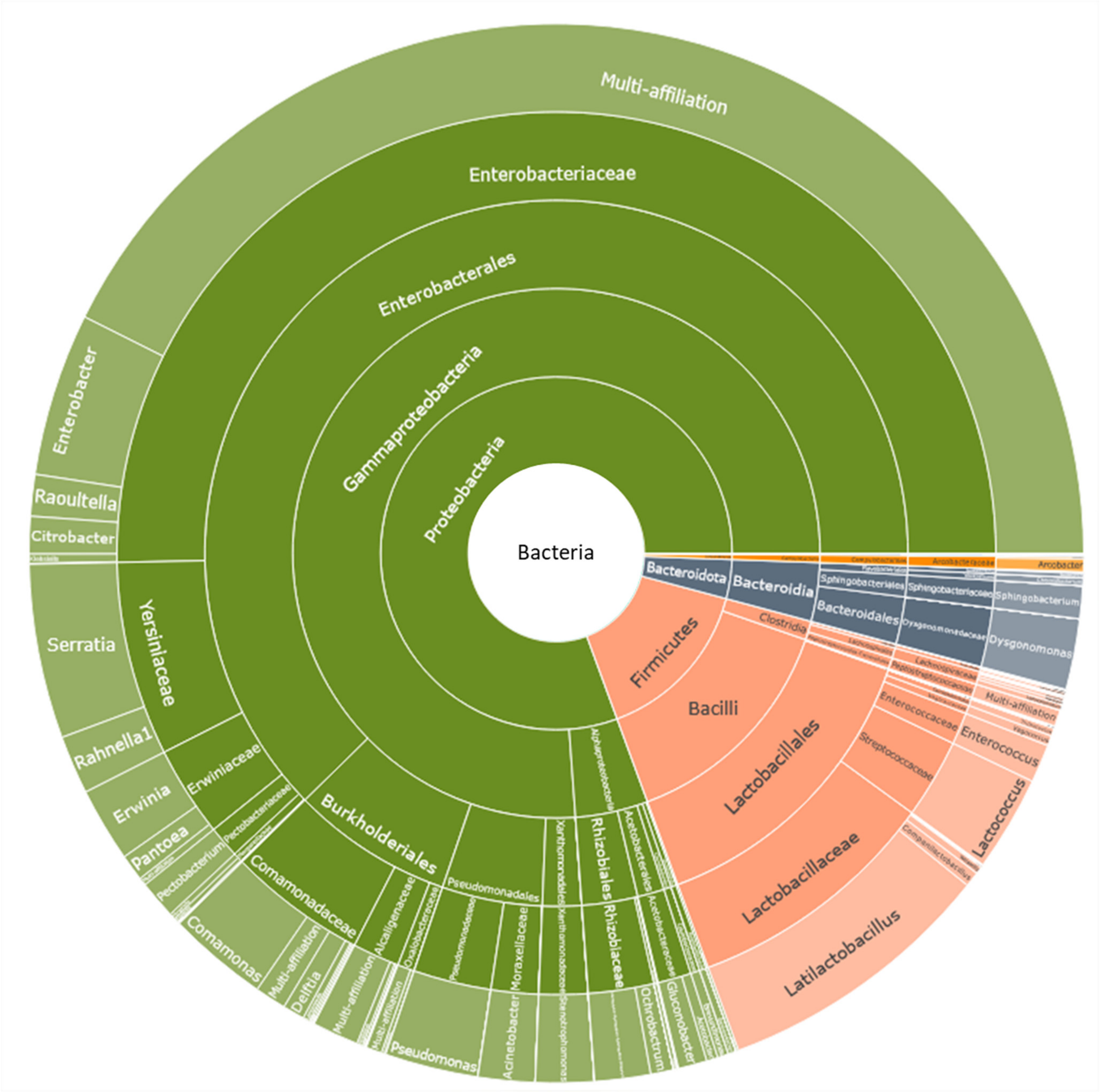

S3-C Taxonomic distribution of microbial community in frass samples (HI_frass_D5, HI_frass_D14).

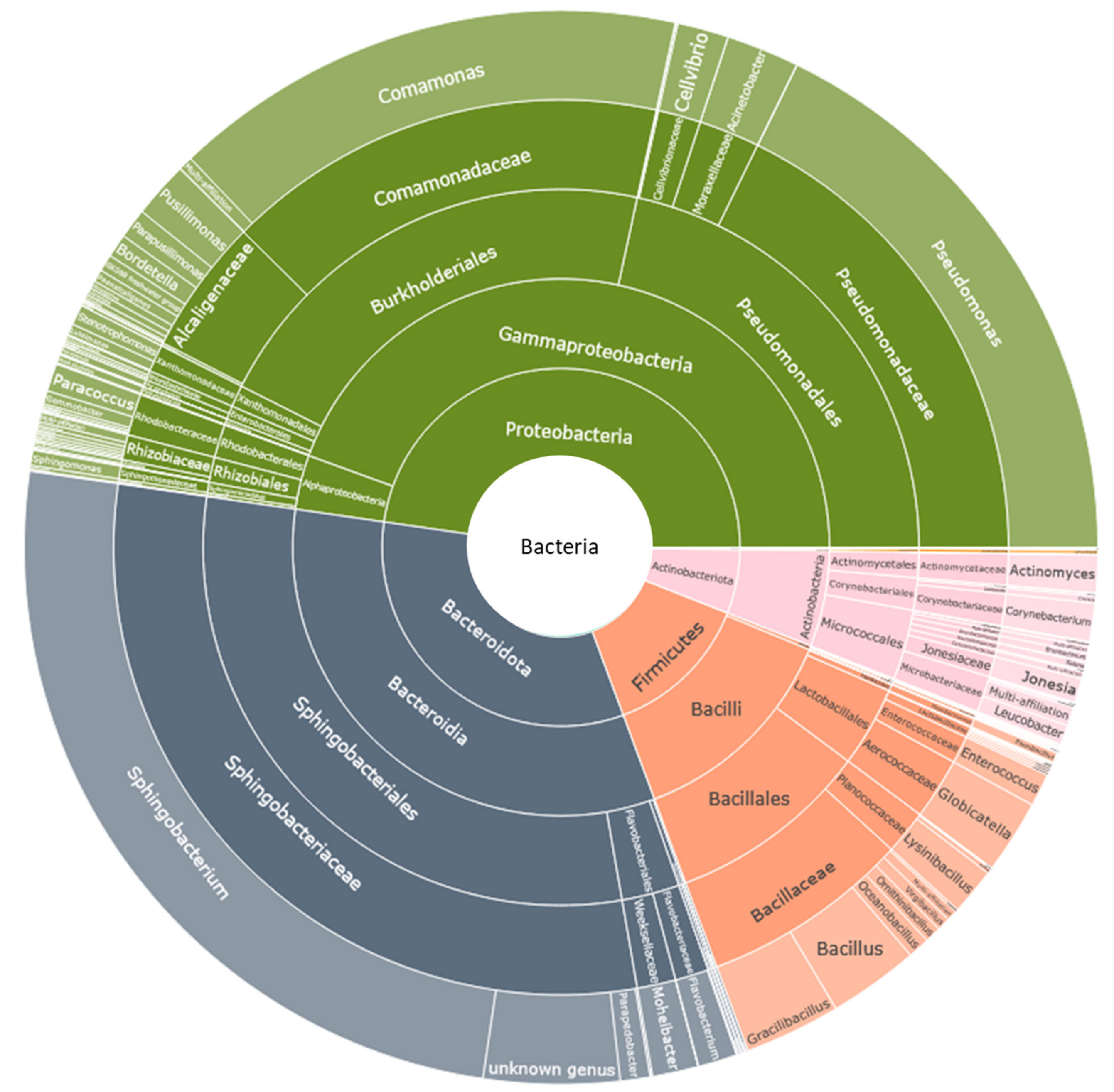

S3-D Taxonomic distribution of microbial community in larvae samples (HI_larvae_D5, HI_larvae_D14, HI_P1_larvae_D14_defrosted, HI_P2_larvae_D14_defrosted).

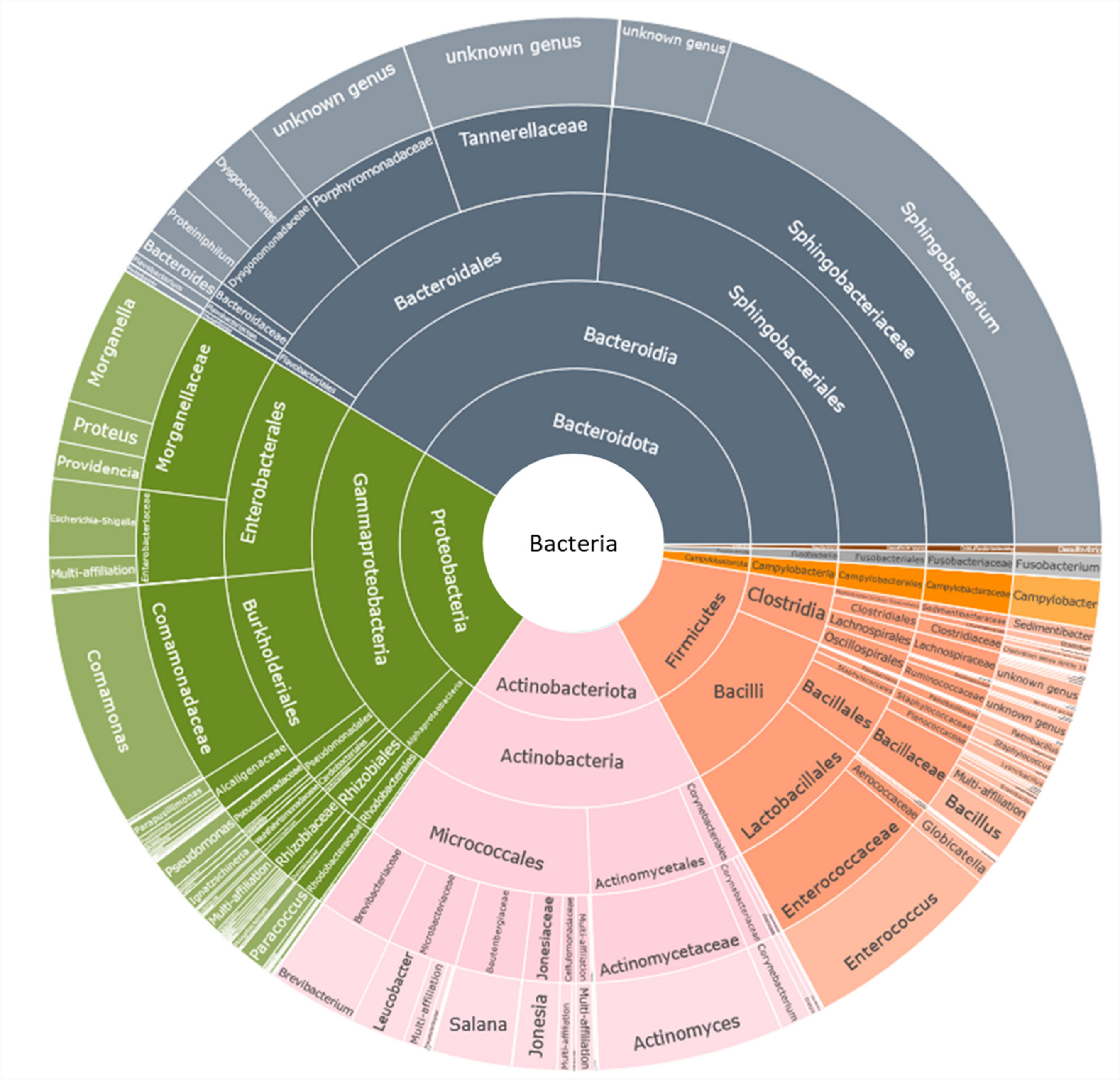

S3-E Taxonomic distribution of microbial community in processed larvae samples (HI_P1_larvae_D14_boiled).

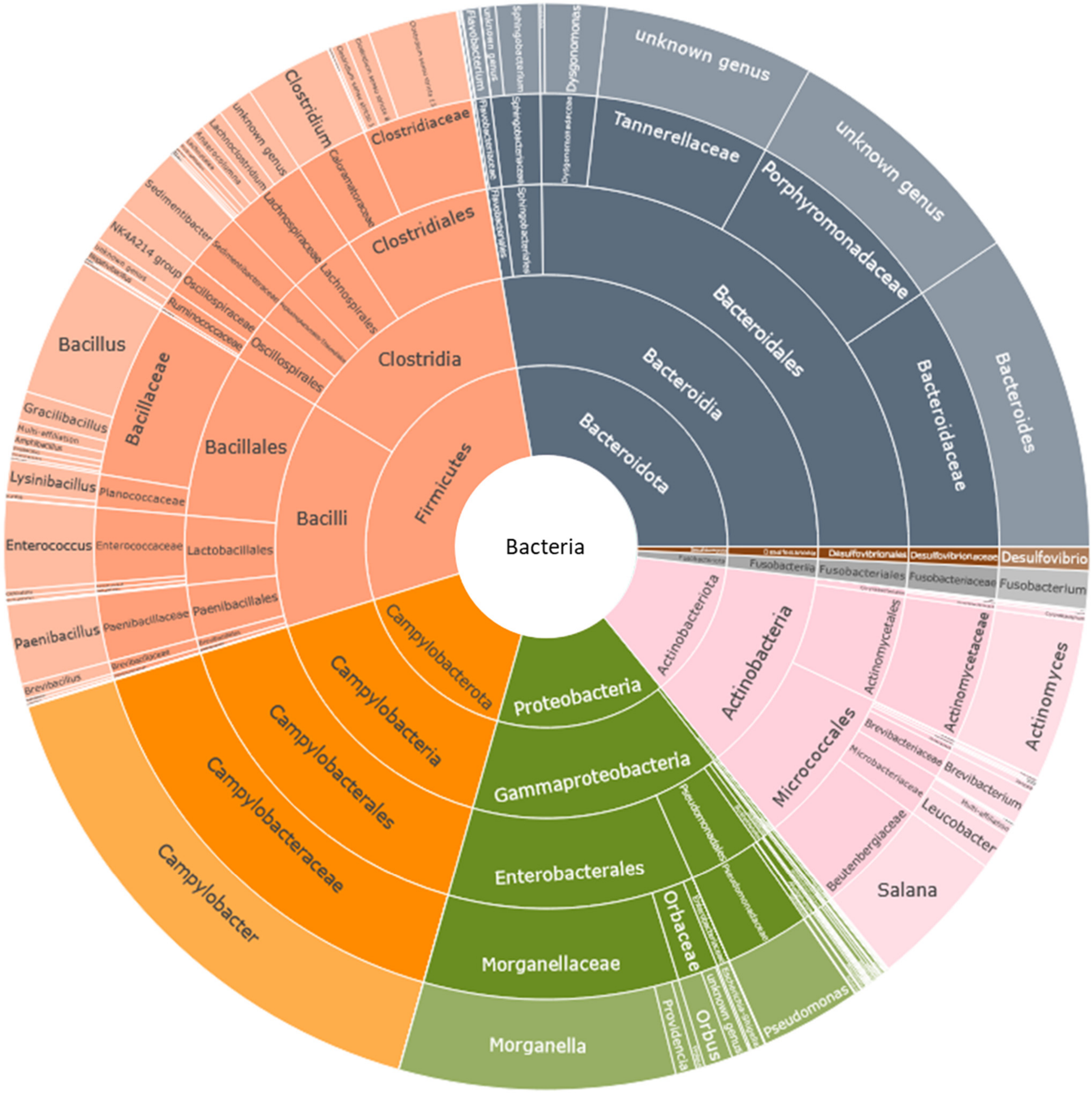

S3-F Taxonomic distribution of microbial community in powder samples (HI_P1_powder_D0, HI_P1_powder_M3, HI_P2_powder_D0, HI_P2_powder_M3).

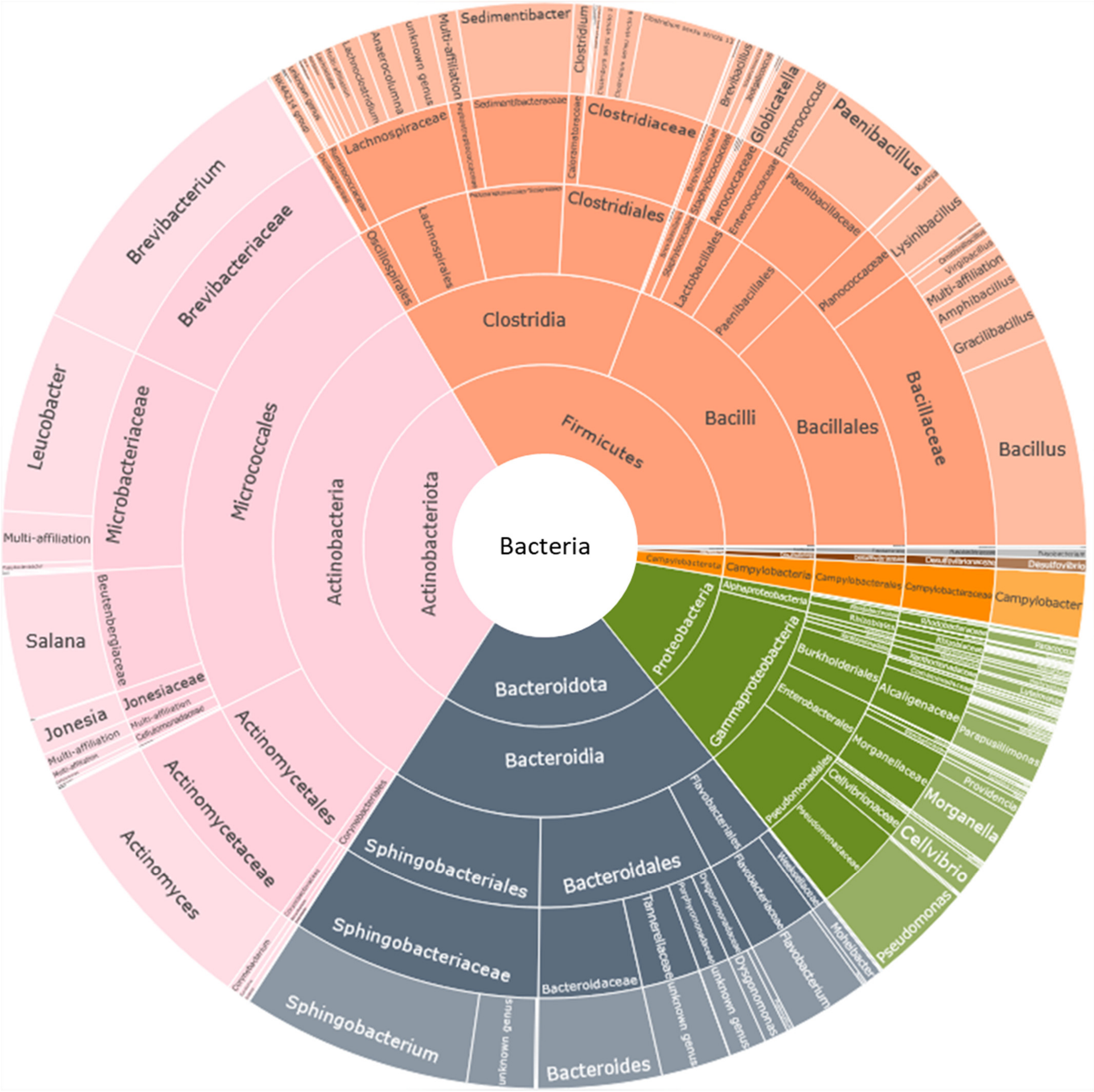

Supplement: Supplementary file 1 [file foods-14-02161-s001.zip › Supplementary file S3.pdf]
