# Supplementary material for: Investigating the Microbial Dynamics of Hermetia illucens Powder Throughout Rearing and Processing: An Integrated Approach Using Cultural and Metabarcoding Methods
Source: Foods. 2025 Jun 20;14(13):2161. doi: 10.3390/foods14132161 (PMC12248505; doi:10.3390/foods14132161)
Supplement: Supplementary file 1 [file foods-14-02161-s001.zip › Supplementary file S4.pdf]

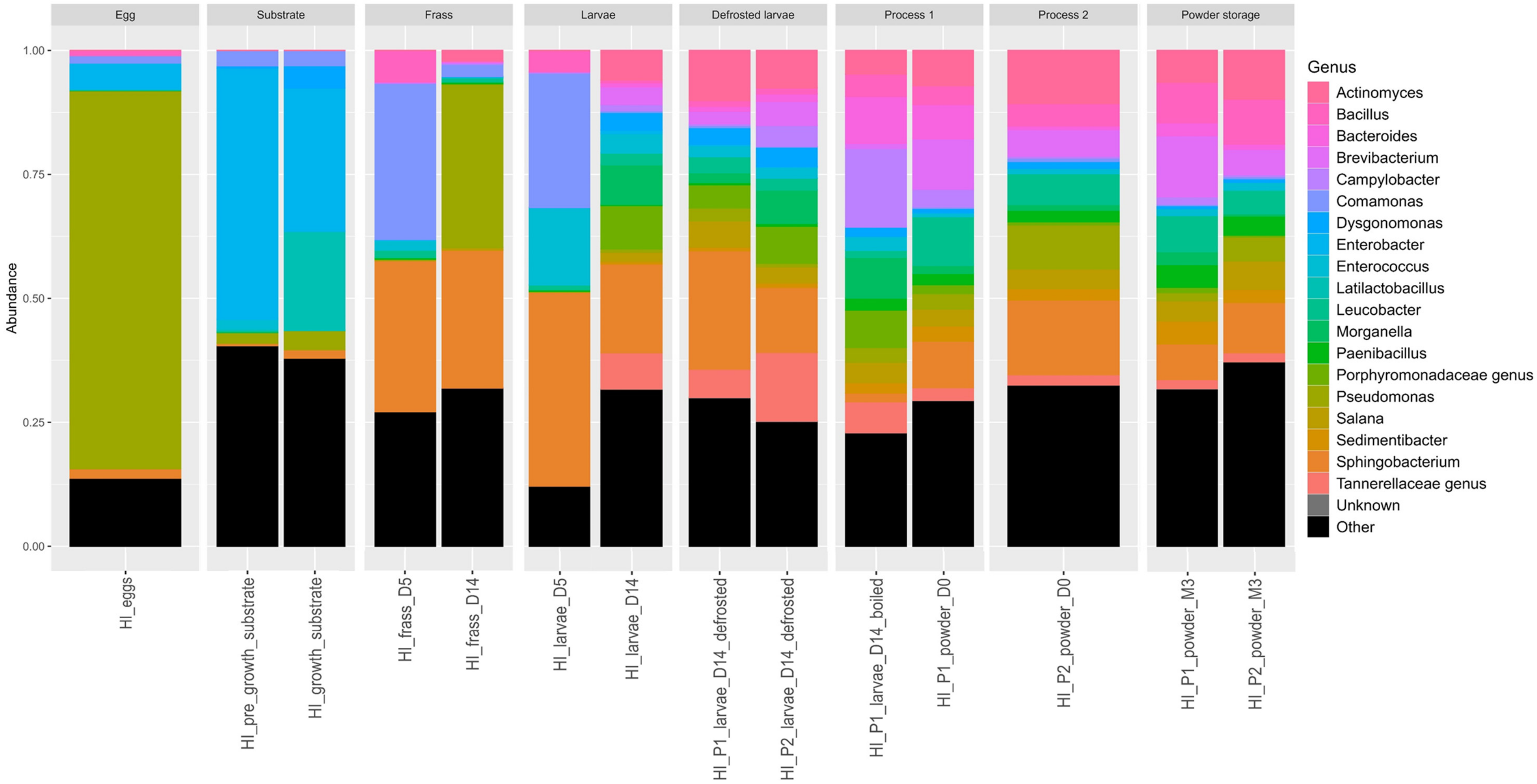

Supplementary File S4. Phylogenetic taxonomy for bacterial communities showing dynamic changes at the genus level during *Hermetia illucens* rearing and processing.
